# Visible Light–Induced Decarboxylative Radical Addition of Heteroaromatic Carboxylic Acids to Alkenes at Room Temperature in Two‐Molecule Photoredox System

**DOI:** 10.1002/open.202500232

**Published:** 2025-04-28

**Authors:** Daisuke Suzuki, Ryoga Hashimoto, Toshiki Furutani, Mugen Yamawaki, Hirotsugu Suzuki, Yasuharu Yoshimi

**Affiliations:** ^1^ Department of Applied Chemistry and Biotechnology Graduate School of Engineering University of Fukui 3‐9‐1 Bunkyo Fukui 910‐8507 Japan; ^2^ Department of Chemistry and Biology Fukui College National Institute of Technology Genshi‐cho Fukui 916‐8507 Japan

**Keywords:** biphenyls and dicyanoanthracenes, heteroaromatic carboxylic acids, photoinduced decarboxylations, room temperatures, two‐molecule photoredox systems

## Abstract

The photoinduced decarboxylative radical addition of aryl carboxylic acids, including heteroaromatic carboxylic acids, to electron‐deficient alkenes is achieved in a two‐molecule photoredox system using a combination of biphenyl (**BP**) and 9,10‐dicyanoanthracene (**DCA**) without heating. The low efficiency of the back‐electron transfer to aryl carboxyl radicals leads to decarboxylation at room temperature. Various heteroaromatic carboxylic acids, including picolinic acid, nicotinic acid, quinoline carboxylic acid, and pyridazine carboxylic acid, are employed as substrates in the photoreaction. Prolonged irradiation without activation by a base successfully leads to decarboxylation by promoting the deprotonation of carboxylic acids by **BP** ^•+^.

## Graphical abstract

The photoinduced decarboxylative radical addition of aryl carboxylic acids, including heteroaromatic carboxylic acids, to electron‐deficient alkenes is achieved in a two‐molecule photoredox system using a combination of biphenyl and 9,10‐dicyanoanthracene without heating or base.

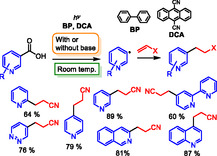

## 1 Introduction

**Scheme 1:**
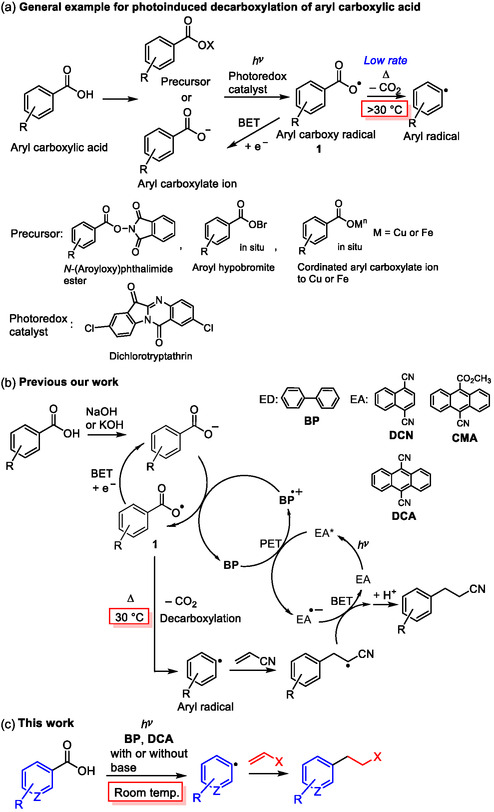
a) General example of photoinduced decarboxylation of aryl carboxylic acid. b) Our previous study. c) Current study.

Photoinduced decarboxylative radical functionalization of aryl carboxylic acids through the formation of aryl radicals under milder conditions than used for transition metal–catalyzed decarboxylation (>140 °C)^[^ ^1^ ^]^ has recently emerged as a research topic in organic synthesis,^[^ ^2^ ^]^ driven by the widespread occurrence of aryl carboxylic acids in nature, in addition to their inexpensiveness and facile storage and handling. However, the lower rate (*k* = 10^6^ s^−1^) of decarboxylation of aryl carboxy radical **1** compared to that (*k* = 10^9^ s^−1^) of alkyl carboxy radicals requires an ingenious method, such as preparation of the precursor from aryl carboxylic acids,^[^ ^3^ ^]^ generation of the precursor in situ,^[^ ^4^ ^]^ the use of excess amounts (>2.5 eq.) of transition metals (Cu^[^ ^5^ ^]^ or Fe^[^ ^6^ ^]^) with oxidants, or the use of Co oxidant^[^ ^7^ ^]^ and mild heating (>30 °C)^[^ ^3^, ^4^, ^5^, ^6^ ^]^ (**Scheme** **1a**). Very recently, the transition metal–free photoinduced decarboxylation of benzoic acids using an organic photoredox catalyst such as dichlorotryptathrin through the formation of an electron‐donor‐acceptor (EDA) complex was successfully achieved.^[^ ^8^ ^]^ However, the preparation of dichlorotryptathrin and heating (35–40 °C) were required. Thus, direct photoinduced decarboxylative modification of aryl carboxylic acids without the preparation of precursors, photoredox catalysts, transition metals, or heating is desirable.

We previously described the direct photoinduced decarboxylative radical reactions of aryl carboxylic acids in a two‐molecule photoredox system^[^ ^9^ ^]^ using a combination of biphenyl (**BP**) as an electron donor (ED) and 1,4‐dicyanonaphthalene (**DCN**)^[^ ^10^ ^]^ or 9,10‐dicyanoanthracene (**DCA**)^[^ ^10^ ^]^ or 9‐cyano‐10‐methoxycarbonylanthracene (**CMA**)^[^ ^11^ ^]^ as an electron acceptor (EA) by irradiation with UV (313 nm) or visible light (405 nm) with mild heating (30 °C) (Scheme 1b). This process is initiated by photoinduced electron transfer (PET) between **BP** and EA to generate **BP** ^•+^, which oxidizes the aryl carboxylate ion to form **1**. The two‐molecule photoredox system promoted the decarboxylation of **1** with mild heating to generate aryl radicals, owing to the low efficiency of back‐electron transfer (BET) from EA^•−^. The generated aryl radicals reacted with radical‐trapping reagents such as acrylonitrile to furnish the adduct in high yield through BET and protonation. By contrast, the one‐molecule photoredox system using the Ir or Fukuzumi catalyst was unsuccessful,^[^ ^10^ ^]^ and the starting material was nearly completely recovered because of the low rate of decarboxylation of **1** and the high rate of BET from the reductant part of the Ir and Fukuzumi catalysts. Thus, suppressing competitive BET from the reductant of the photoredox catalyst to **1** is important for the successful photoinduced decarboxylation of aryl carboxylic acids. In fact, the photoinduced reduction of the prepared *N*‐(aroyloxy)phthalimide esters^[^ ^3^ ^]^ or benzoyl hypobromites^[^ ^4^ ^]^ generated in situ (Scheme 1a) by the photoredox catalysts led to efficient decarboxylation because of the absence of the reductant of the photoredox catalysts, which precluded interruption of the BET to **1**.

Based on this, the photochemical conditions were screened using two‐molecule photoredox catalysts for the milder decarboxylation of aryl carboxylic acids. Careful investigation revealed that the photoinduced decarboxylative radical addition of aryl carboxylic acids to alkenes proceeded well when the **BP**/**DCA** system was used under visible light irradiation at room temperature (RT), which is also applicable to heteroaromatic carboxylic acids (Scheme 1c). In addition, photoinduced decarboxylation in the absence of a base furnished the adducts in moderate yields. These results showed that the photoinduced radical decarboxylation of aryl carboxylic acids does not require transition metals and heating when the suitable combination of photoredox catalysts ED/EA were selected in the two‐molecule photoredox system, which led to the large benefit for modification of aryl carboxylic acids.

## 2 Results and Discussion

Initially, attempts were made to optimize the combination of ED/EA and the reaction conditions for the photoinduced decarboxylative radical addition at RT using benzoic acid **2a** (20 mM), base (NaOH or KOH 20 mM), and acrylonitrile **3 A** (100 mM) in an aqueous acetonitrile solution (CH_3_CN/H_2_O = 9:1, v/v) (**Table** **1**). As reported by the authors for aryl radical reactions, competitive aryl radical H abstraction from CH_3_CN and addition to **3 A** occurred simultaneously during the photoreaction, and a high concentration of **3 A** (5 equiv.) was necessary.^[^ ^12^ ^]^

**Table 1:** Visible light–induced decarboxylative radical addition of **2a** to **3A** using ED and **DCA**.

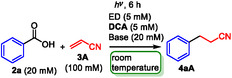				
Entry	ED	Base	Yield of **4aA**	Recovery of **2a**
1	**BP**	NaOH	70%	0%
2	–	KOH	71%	0%
3a)	–	–	65%	0%
4	**NA**	NaOH	3%	84%
5	–	KOH	4%	81%
6	**Phen**	NaOH	10%	66%
7	–	KOH	11%	68%
8	None	–	5%	82%
9	**BP**	None	21% (56%)b)	49% (0%)^b)^
10c)	–	–	0%	Quantitative

a) At 30 °C.

b) Irradiation time of 24 h.

c) [HCl] = 100 mM.

In the case of **BP**/**DCN** with irradiation at 313 nm or **BP**/**CMA** with irradiation using a 405 nm light‐emitting diode (LED) in the presence of NaOH, a lower yield of the adduct **4aA** (**BP**/**DCN**: 69–20%, **BP**/**CMA**: 80–45%) was obtained when the temperature was decreased from 30 °C to ≈20 °C (RT) using a water bath (Table S1, Supporting Information), as previously reported,^[^ ^10^, ^11^ ^]^ except when **BP**/**CMA** was utilized at RT. Fortunately, in the presence of a base (KOH or NaOH) at RT, the **BP**/**DCA** system provided a good yield of **4aA** (70% or 71%) after 6 h under irradiation with a 405 nm LED (entries 1 and 2), along with no recovery of **2a**. A slightly lower yield of **4aA** (65%) was obtained at 30 °C owing to the promotion of radical oligomerization (entry 3). **DCA** (5 mM) was not completely soluble in the aqueous acetonitrile solution, where the solubility of **DCA** (less than 0.3 mM) was determined from the UV absorption of **DCA** after filtration. However, **DCA** concentrations higher than 0.3 mM led to an increase in the yield of **4aA** by providing sufficient **DCA** to prevent a decrease in the concentration. The use of naphthalene (**NA**, oxidation potential of **NA**: +1.70 V vs saturated calomel electrode (SCE) in CH_3_CN)^[^ ^13^ ^]^ or phenanthrene (**Phen**, oxidation potential of **Phen**: +1.50 V vs SCE in CH_3_CN)^[^ ^13^ ^]^ as the ED instead of **BP** (oxidation potential of **BP**: +1.95 V vs SCE in CH_3_CN)^[^ ^14^ ^]^ led to low efficiency of the photoinduced decarboxylation (entries 4–7), and the counter‐cation (K^+^ or Na^+^) was not affected, unlike the arylborate ions.^[^ ^15^ ^]^ As reported by the authors,^[^ ^16^ ^]^ both a lower concentration of EA (**DCA**) and a smaller negative Δ*G*_PET_ between the ED and EA led to a lower concentration of EA^•−^ and decreased the efficiency of BET (Δ*G*_PET_ between **BP** and **DCA**: +2.72 kJ mol^−1^, Δ*G*_PET_ between **NA** and **DCA**: −21.4 kJ mol^−1^, Δ*G*_PET_ between **Phen** and **DCA**: −40.7 kJ mol^−1^). Thus, the **BP**/**DCA** combination could promote the decarboxylation of **1** even at RT, because of its lower BET efficiency.

**Scheme 2:**
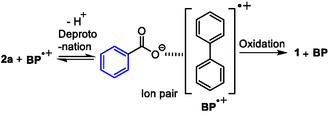
Formation of an ion pair between the benzoate ion and **BP** ^•+^.

The absence of **BP** significantly decreased the yield of **4aA** (5%) because of the high BET efficiency, indicating that a two‐molecule photoredox system is essential for the efficient photoinduced decarboxylation of **2a** (entry 8). Although the absence of a base decreased the yield **4aA** (21%), prolonged irradiation (24 h) resulted in moderate yield of **4aA** (56%, entry 9 in parentheses). This is why **BP** ^•+^ promotes the deprotonation of **2a** to generate an ion pair consisting of a benzoate ion and **BP** ^•+^ (**Scheme** **2**), which has been previously observed in the similar two‐molecule photoredox reaction of **2a**.^[^ ^17^ ^]^ Furthermore, the presence of acids such as HCl (100 mM) prevented deprotonation by **BP** ^•+^, which disturbs decarboxylation, along with the complete recovery of **2a** (entry 10), because **BP** ^•+^ can't oxidize **2a** (PhCO_2_H, oxidation potential of **2a**: +2.06 V vs SCE in CH_3_CN),^[^ ^18^ ^]^ but can oxidize the carboxylate ion of **2a** (PhCO_2_^−^, oxidation potential of the carboxylate ion of **2a**: +1.40 V vs SCE in CH_3_CN).^[^ ^18^ ^]^ Thus, the direct decarboxylative functionalization of **2a** without activation by a base was achieved, even though a longer irradiation time was required.

The scope and limitations of the aromatic carboxylic acids **2** in the photoreaction of **3 A** at RT were next explored. Although the irradiation time was dependent on the type of aromatic carboxylic acid, the photoreactions of benzoic acids **2b**,**c** bearing electron‐donating or electron‐withdrawing groups and *N*‐heteroaromatic carboxylic acids such as picolinic acid **2d**, nicotinic acid **2e**, isonicotinic acid **2f**, bipyridyl carboxylic acid **2g**, quinoline carboxylic acids **2h–j**, isoquinoline carboxylic acid **2k**, pyridazine carboxylic acid **2l **, and pyrimidine carboxylic acid **2m** led to moderate to good yields of adducts **4bA–mA** under the optimized conditions. Because of the low solubility of coumarine carboxylic acid **2n** in aqueous acetonitrile, a long irradiation time and slight stirring provided adduct **4nA**. As mentioned later, acridine carboxylic acid **2o** slightly disturbed the light absorption of **DCA** and mainly disturbed PET between **BP** and **DCA**, resulting in a low yield of adduct **4oA** under prolonged irradiation. By contrast, heteroaromatic carboxylic acids with furane **2p**,**q**, thiophene **2r**,**s**, pyrrole **2 t**, indole **2u**,**v**, and isoquinoline **2w** provided a complex mixture because the direct photoredox reaction of the heteroaromatic rings occurred under these photochemical conditions. In addition, the suppression of PET between **BP** and **DCA** by aromatic carboxylic acid **2x–ac** and/or the decarboxylation of **1** from **2x–ac** completely recovered the carboxylic acid **2x–ac**. When **2b**,**c**,**e**,**h** were subjected to the photoreaction in the absence of a base under prolonged irradiation, moderate yields of adducts **4bA**,**cA**,**hA** were obtained, except for **4eA** (in parentheses in **Table** **2**). The reason for the difference in the reactivity in the presence/absence of a base is not clear yet, but various heteroaromatic carboxylic acids were employed in the photoinduced decarboxylative addition of **3A** with a base.

**Table 2:** Scope and limitations of aromatic carboxylic acid **2** in the photoinduced decarboxylative radical addition to **3 A**.a), b), c), d), e), f)

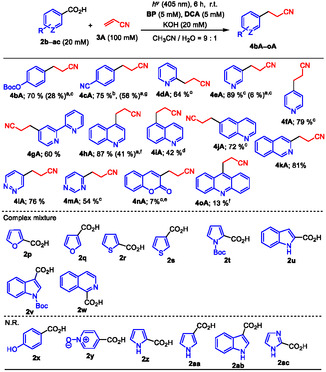

a) Without base.

b) Irradiation time was 9 h.

c) Irradiation time was 24 h.

d) Irradiation time was 36 h.

e) Stirring.

f) Irradiation time was 48 h.

g) Irradiation time was 60 h.

The use of relatively poorly electron‐deficient alkenes, such as acrylic acid esters **3B** and **C**, vinyl sulfone **3D**, and acrylamides **3E** and **F** instead of **3A** in the photoreaction of **2e** for 24 h at RT, resulted in the formation of the corresponding adducts **4eB**–**eF** in lower yields due to radical oligomerization of the relatively poorly electron‐deficient alkenes (**Table** **3**).^[^ ^19^ ^]^

**Table 3:** Scope of electron‐deficient alkenes **3** in the photoinduced decarboxylative radical addition of **2e**.

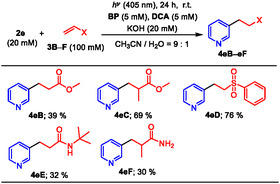

**Figure 1:**
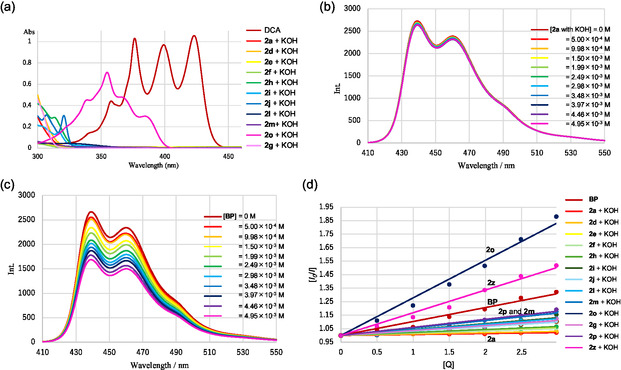
a) UV−vis absorption spectra of **DCA** and **2** with 1 equiv. KOH (1 × 10^−4^ m) in aqueous acetonitrile (CH_3_CN/H_2_O = 9:1, v/v), and b) fluorescence quenching of **DCA** (1 × 10^−4^ m) by **2a** with 1 equiv. KOH and c) after excitation of **BP** at 405 nm in aqueous acetonitrile (CH_3_CN/H_2_O = 9:1, v/v). d) Stern–Volmer plots.

The UV–vis absorption spectra of **DCA** and **2** with 1 equiv. KOH indicated that **DCA** was excited only at 405 nm, unlike **2o** (**Figure** **1**a). The fluorescence of **DCA** was slightly quenched by **2a** in the presence of 1 equiv. KOH (Figure 1b) and was relatively strongly quenched by **BP** (Figure 1c). Stern–Volmer plots of the fluorescence quenching of **DCA** by **BP** or **2** with 1 equiv. of KOH showed that the rate of PET between **DCA** and **BP** was higher than that between **DCA** and **2** with 1 equiv. KOH, except in the case of **2o** and **2z** (Figure 1d). The fluorescence quenching of **DCA** by **2** with KOH and the method for calculation of *k*_q_ are shown in Figure S1, Supporting Information. The suppression of PET between **BP** and **DCA** requires a long irradiation time (**2o**) or causes no reaction, with the complete recovery of **2z**. Additionally, the relatively high rate of PET between **DCA** and **2p** provided a complex mixture. These results indicate that the initial process in the successful photoinduced decarboxylation was PET between **BP** and **DCA** to generate **BP** ^•+^ and **DCA** ^•−^, and that **BP** ^•+^ oxidized the carboxylate ion of **2** to furnish adduct **4**, as shown in Scheme 1b.

## 3 Conclusion

Photoinduced decarboxylative radical addition of heteroaromatic carboxylic acids **2** to alkenes **3** using **BP** and **DCA** at RT was successful. The low efficiency of BET to **1** (Scheme 1b) using the combination of **BP**/**DCA** owing to the low solubility of **DCA** and the relatively small negative Δ*G*_PET_ between **BP** and **DCA** led to the decarboxylation of **1** at RT. These results provided us the detailed insights about mechanism of photoinduced radical decarboxylation of aryl carboxylic acids. Various heteroaromatic carboxylic acids, including picolinic acid, nicotinic acid, isonicotinic acid, quinoline carboxylic acid, isoquinoline carboxylic acid, and pyridazine carboxylic acid, were employed as substrates in the photoreaction, although furane, thiophene, pyrrole, and indole carboxylic acids were not suitable. Even if a long irradiation time is required, **BP** ^•+^ promotes the deprotonation of **2**, causing decarboxylation without activation by the base. Thus, milder photoinduced decarboxylation of **2** using **BP**/**DCA** system without heating or base was achieved.

## 4 Experimental Section

4.1

4.1.1

##### General Procedure for the Photoreaction of 2 with 3

An aqueous CH_3_CN solution (CH_3_CN 18.0 mL, H_2_O 2.0 mL) of aryl carboxylic acid **2** (20 mM, 0.4 mmol), KOH (1 eq., 20 mM, 0.4 mmol, 0.0225g), **BP** (0.25 eq., 5 mM, 0.1 mmol, 0.0154 g), and **DCA** (0.25 eq., 5 mM, 0.1 mmol, 0.0228 g) in Pyrex vessels (10 × 120 mm) was purged with argon for 10 min, and electron‐deficient alkene **3** (5 eq., 100 mM, 2 mmol) was added under argon atmosphere. The mixture was irradiated with a 18 W blue LED (405 nm) at RT using a water bath without stirring, and then the solvent was removed under reduced pressure. The crude product was purified by silica‐gel column chromatography using hexane/EtOAc as the eluents to yield adducts **4**.

## Conflict of Interest

The authors declare no conflict of interest.

## Supporting information

Supplementary Material

## Data Availability

The data that support the findings of this study are available in the supplementary material of this article.
