# Supplementary material for: Visible Light–Induced Decarboxylative Radical Addition of Heteroaromatic Carboxylic Acids to Alkenes at Room Temperature in Two‐Molecule Photoredox System
Source: ChemistryOpen. 2025 Apr 28;14(10):e202500232. doi: 10.1002/open.202500232 (PMC12518024; doi:10.1002/open.202500232)

Supporting Information

Visible-Light-Induced Decarboxylative Radical Addition of Heteroaromatic Carboxylic Acids to Alkenes at Room Temperature in Two-Molecule Photoredox System

Daisuke Suzuki[a], Ryoga Hashimoto[a], Toshiki Furutani[a,b], Mugen Yamawaki[b], Hirotsugu Suzuki[a], Yasuharu Yoshimi[a,*]

[a]Department of Applied Chemistry and Biotechnology, Graduate School of Engineering, University of Fukui, 3-9-1 Bunkyo, Fukui 910-8507, Japan

[b]Department of Chemistry and Biology, National Institute of Technology, Fukui College, Geshi-cho, Fukui 916-8507, Japan

E-mail of the corresponding author: yyoshimi@u-fukui.ac.jp

Table of Contents

General information · S2

Optimization for photoreaction of 2a with 3A · S2

Fluorescence quenching of DCA by 2 with KOH and k_q · · · · · · · · · · · · · · · · · · S3

Characterization data for photoproduct 4 · S5

References in SI · S10

^{1}H and ^{13}C{^{1}H} NMR Spectra of 4aA · S12

^{1}H and ^{13}C{^{1}H} NMR Spectra of 4bA · S13

^{1}H and ^{13}C{^{1}H} NMR Spectra of 4cA · S14

^{1}H and ^{13}C{^{1}H} NMR Spectra of 4dA · S15

^{1}H and ^{13}C{^{1}H} NMR Spectra of 4eA · S16

^{1}H and ^{13}C{^{1}H} NMR Spectra of 4fA · S17

^{1}H and ^{13}C{^{1}H} NMR Spectra of 4gA · S18

^{1}H and ^{13}C{^{1}H} NMR Spectra of 4hA · S19

^{1}H and ^{13}C{^{1}H} NMR Spectra of 4iA · S20

^{1}H and ^{13}C{^{1}H} NMR Spectra of 4jA · S21

^{1}H and ^{13}C{^{1}H} NMR Spectra of 4kA · S22

^{1}H and ^{13}C{^{1}H} NMR Spectra of 4lA · S23

^{1}H and ^{13}C{^{1}H} NMR Spectra of 4mA · S24
^{1}H and ^{13}C{^{1}H} NMR Spectra of 4nA · S25
^{1}H and ^{13}C{^{1}H} NMR Spectra of 4oA · S26
^{1}H and ^{13}C{^{1}H} NMR Spectra of 4eB · S27
^{1}H and ^{13}C{^{1}H} NMR Spectra of 4eC · S28
^{1}H and ^{13}C{^{1}H} NMR Spectra of 4eD · S29
^{1}H and ^{13}C{^{1}H} NMR Spectra of 4eE · S30
^{1}H and ^{13}C{^{1}H} NMR Spectra of 4eF · S31

General information. All reagents and solvents including **2** and **3** were used as received from commercial suppliers. IR spectra were recorded on an FT-IR spectrometer. ^{1}H NMR spectra were recorded in $CDCl_3$ containing tetramethylsilane as an internal standard, and were acquired on a 500 MHz spectrometer. ^{13}C{^{1}H} NMR spectra were acquired on a 125 MHz spectrometer. High-resolution mass spectra were obtained using DART mass spectrometer. The visible light source was a 18 W blue LED (EvoluChem, P206-18-1 405 nm). Pyrex vessels (10 mm × 120 mm) were set at a distance of 3 cm from the LED in a water-bath. Column chromatography was performed on a Wakogel C-300 instrument with a particle size of 45−75 μm. Photoproducts **4aA−4fA** and **4eB** were already reported.[S1−S4]

Optimization for photoreaction of 2a with 3A

In the case of **BP/DCN** with irradiation at 313 nm (entries 1 and 2 in Table S1) or **BP/CMA** with irradiation using a 405 nm LED (entries 3 and 4) in the presence of NaOH, a lower yield of the adduct **4aA** (**BP/DCN**: 69 to 20%, **BP/CMA**: 80 to 45%) was obtained when the temperature was decreased from 30 °C to ca. 20 °C (room temperature) using a water-bath. Fortunately, in the presence of a NaOH at room temperature, the **BP/DCA** system provided a good yield of **4aA** (70%) with a 405 nm LED (entry 6), along with no recovery of **2a**. A slightly lower yield of **4aA** (65%) was obtained at 30 °C owing to the promotion of radical oligomerization (entry 5).

Table S1. Photoreaction of **2a** with **3A** in the presence of ED/EA at 30 °C or room temperature

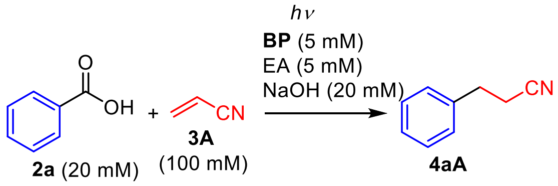

Entry	Light source	Temperature	EA	Yield of **4aA**/%
1	UV (313 nm)	30 °C	**DCN**	69
2		r. t.		20
3	Visible (405 nm)	30 °C	**CMA**	80
4		r. t.		45
5	Visible (405 nm)	30 °C	**DCA**	65
6		r. t.		70

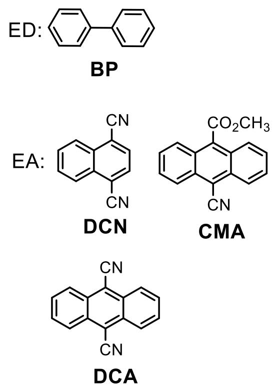

Fluorescence quenching of DCA by 2 with KOH and *k*q.

Fluorescence quenching of **DCA** (1×10^{-4} M) by **2** with 1 eq. KOH excited at 405 nm in an aqueous acetonitrile solution (CH_3CN/H_2O=9:1, *v*/*v*) was shown in Figure S1.

(a) Fluorescence quenching of **DCA** by **2d** with KOH

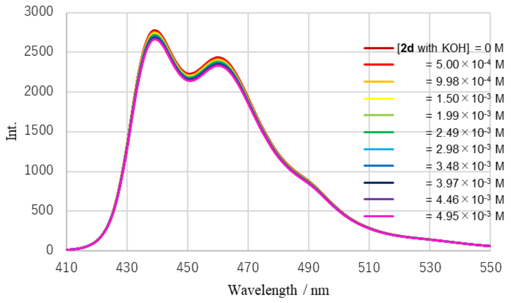

(b) Fluorescence quenching of **DCA** by **2e** with KOH

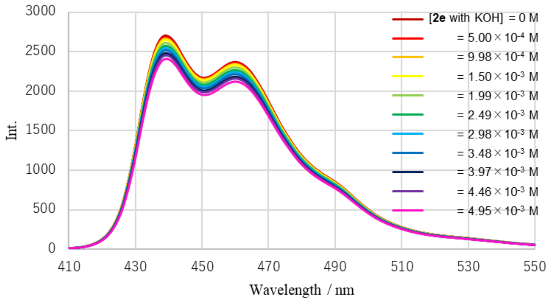

(c) Fluorescence quenching of **DCA** by **2f** with KOH

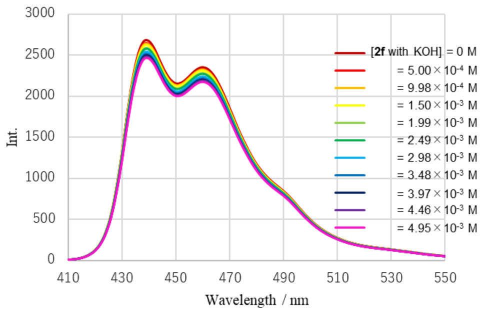

(d) Fluorescence quenching of **DCA** by **2h** with KOH

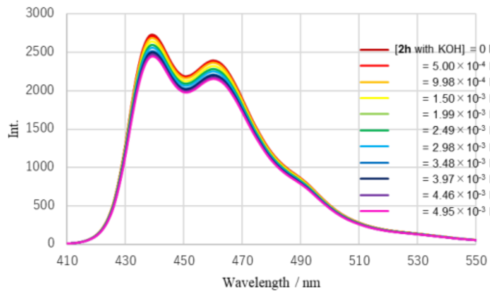

(e) Fluorescence quenching of **DCA** by **2i** with KOH

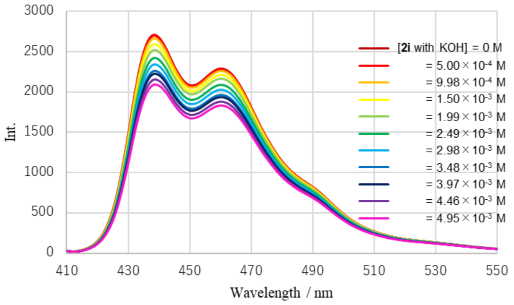

(f) Fluorescence quenching of **DCA** by **2j** with KOH

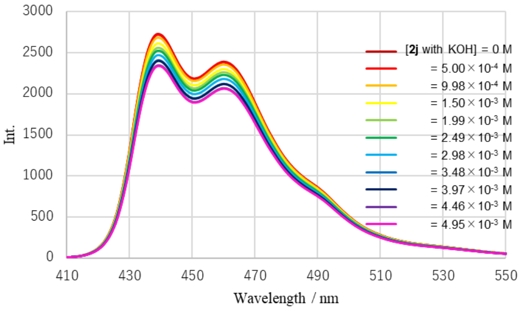

(g) Fluorescence quenching of **DCA** by **2l** with KOH

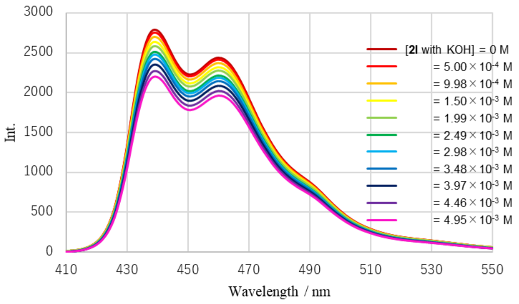

(h) Fluorescence quenching of **DCA** by **2m** with KOH

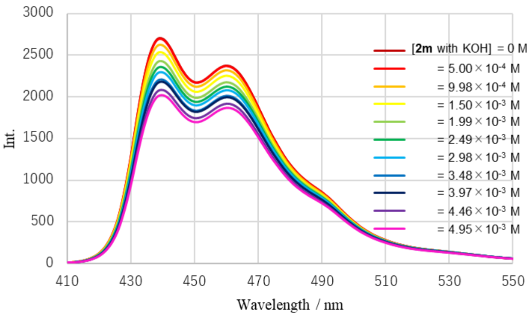

(i) Fluorescence quenching of **DCA** by **2o** with KOH

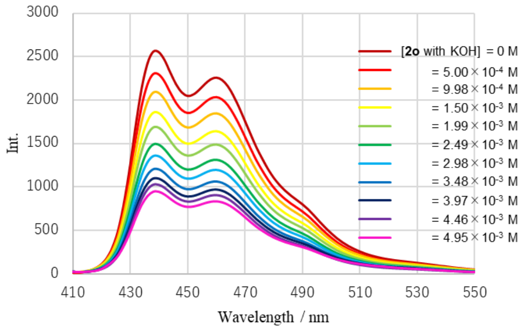

(j) Fluorescence quenching of **DCA** by **2g** with KOH

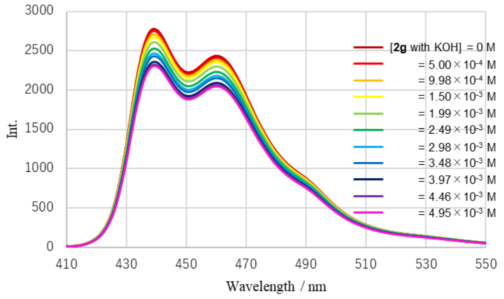

(k) Fluorescence quenching of **DCA** by **2p** with KOH

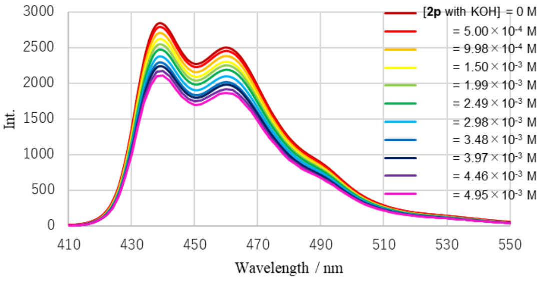

(l) Fluorescence quenching of **DCA** by **2z** with KOH

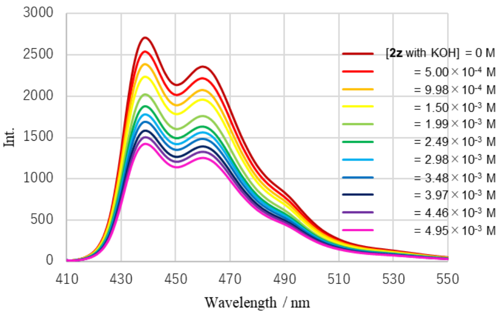

Figure S1. Fluorescence quenching of **DCA** (1×10^{-4} M) by **2** with 1 eq. KOH excited at 405 nm in an aqueous acetonitrile solution (CH_3CN/H_2O=9:1, *v*/*v*).

The rate constant for fluorescence quenching (k_q) of **DCA** by **BP** or **2** with 1 eq. KOH was calculated from the Stern-Volmer plots using equation $I_0/I = 1 + k_q\tau[Q]$ (I_0; fluorescence intensity of **DCA** at 438 nm, I; observed fluorescence intensity of **DCA** at 438 nm with quencher, τ =15.1 ns; fluorescence lifetime of **DCA**, [Q]; concentration of quencher).

BP; $k_q = 7.29 \times 10^9$ $M^{-1}s^{-1}$
2a + KOH; $k_q = 4.83 \times 10^8$ $M^{-1}s^{-1}$
2d + KOH; $k_q = 5.96 \times 10^8$ $M^{-1}s^{-1}$
2e + KOH; $k_q = 1.44 \times 10^9$ $M^{-1}s^{-1}$
2f + KOH; $k_q = 1.20 \times 10^9$ $M^{-1}s^{-1}$
2h + KOH; $k_q = 1.52 \times 10^9$ $M^{-1}s^{-1}$
2i + KOH; $k_q = 3.54 \times 10^9$ $M^{-1}s^{-1}$
2j + KOH; $k_q = 2.32 \times 10^9$ $M^{-1}s^{-1}$
2l + KOH; $k_q = 3.17 \times 10^9$ $M^{-1}s^{-1}$
2m+ KOH; $k_q = 4.15 \times 10^9$ $M^{-1}s^{-1}$
2o + KOH; $k_q = 2.12 \times 10^{10}$ $M^{-1}s^{-1}$
2g + KOH; $k_q = 2.72 \times 10^9$ $M^{-1}s^{-1}$
2p + KOH; $k_q = 4.38 \times 10^9$ $M^{-1}s^{-1}$
2z + KOH; $k_q = 1.16 \times 10^{10}$ $M^{-1}s^{-1}$

Characterization data for photoproduct 4.

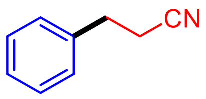

4aA; Compound **4aA** has been previously reported.[S1] using hexane/EtOAc = 4:1 as the eluent, 37.2 mg, 71%, colorless oil, ^{1}H NMR (500 MHz, $CDCl_3$) δ 7.36–7.23 (m, 5H), 2.97 (t, *J* = 7.4 Hz, 2H), 2.63 (t, *J* = 7.4 Hz, 2H); $^{13}C\{^1H\}$ NMR (125 MHz, $CDCl_3$) δ 138.2, 129.0, 128.4, 127.3, 119.3, 31.6, 19.5.

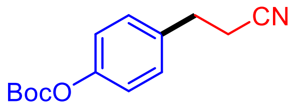

4bA; Compound **4bA** has been previously reported.[S1] using hexane/EtOAc = 4:1 as the eluent, 69.2

mg, 70%, colorless oil, ^{1}H NMR (500 MHz, $CDCl_3$) δ 7.24 (d, J = 8.5 Hz, 2H), 7.14 (d, J = 8.5 Hz, 2H), 2.97 (t, J = 7.4 Hz, 2H), 2.62 (t, J = 7.4 Hz, 2H), 1.57 (s, 9H); ^{13}C{^{1}H} NMR (125 MHz, $CDCl_3$) δ 151.9, 150.3, 135.6, 129.4, 121.8, 119.1, 83.8, 31.1, 27.8, 19.5.

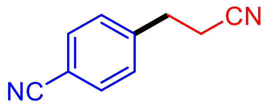

4cA; Compound **4cA** has been previously reported.[S1] using hexane/EtOAc = 4:1 as the eluent, 46.9 mg, 75%, white solid, ^{1}H NMR (500 MHz, $CDCl_3$) δ 7.66 (d, J = 8.0 Hz, 2H), 7.37 (d, J = 8.0 Hz, 2H), 3.03 (t, J = 7.2 Hz, 2H), 2.67 (t, J = 7.2 Hz, 2H); ^{13}C{^{1}H} NMR (125 MHz, $CDCl_3$) 143.2, 132.8, 129.3, 118.6, 118.4, 111.6, 31.6, 19.0.

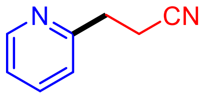

4dA; Compound **4dA** has been previously reported.[S2] using $CHCl_3$/MeOH = 1:0 to 19:1 as the eluent, 37.4 mg, 64%, white solid, ^{1}H NMR (500 MHz, $CDCl_3$) δ 8.56 (d, J = 4.6 Hz, 1H), 7.68−7.64 (m, 1H), 7.23 (d, J = 7.4 Hz, 1H), 7.21−7.19 (m, 1H), 3.13 (t, J = 7.3 Hz, 2H), 2.85 (t, J = 7.3 Hz, 2H); ^{13}C{^{1}H} NMR (125 MHz, $CDCl_3$) δ 157.2, 149.7, 136.9, 123.2, 122.3, 119.6, 33.5, 16.8.

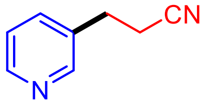

4eA; Compound **4eA** has been previously reported.[S2] using $CHCl_3$/MeOH = 1:0 to 19:1 as the eluent, 52 mg, 89%, colorless oil, ^{1}H NMR (500 MHz, $CDCl_3$) δ 8.56−8.53 (m, 2H), 7.62−7.61 (m, 1H), 7.30 (dd, J = 7.7, 4.9 Hz, 1H), 2.99 (t, J = 7.4 Hz, 2H), 2.66 (t, J = 7.4 Hz, 2H); ^{13}C{^{1}H} NMR (125 MHz, $CDCl_3$) δ 149.8, 149.0, 136.0, 133.5, 123.8, 118.6, 28.9, 19.3.

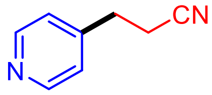

4fA; Compound **4fA** has been previously reported.[S3] using $CHCl_3$/MeOH = 1:0 to 19:1 as the eluent, 45.6 mg, 78%, colorless oil, ^{1}H NMR (500 MHz, $CDCl_3$) δ 8.60−8.58 (m, 2H), 7.22−7.19 (m, 2H), 2.97 (t, J = 7.2 Hz, 2H), 2.68 (t, J = 7.2 Hz, 2H); ^{13}C{^{1}H} NMR (125 MHz, $CDCl_3$) δ 150.5, 150.3, 146.8, 124.2, 123.6, 118.5, 30.8, 18.3.

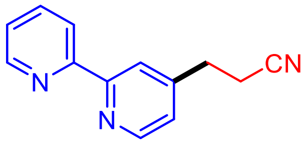

4gA; using $CHCl_3$/MeOH = 1:0 to 19:1 as the eluent, 50.2 mg, 60%, white solid, m.p. 39−40 °C; IR (KBr, cm^{-1}); 2957, 2924, 2246; ^{1}H NMR (500 MHz, $CDCl_3$) δ 8.68 (d, *J* = 5.2 Hz, 1H), 8.65 (d, *J* = 5.2 Hz, 1H), 8.41 (d, *J* = 8.0 Hz, 1H), 8.29 (s, 1H), 7.85−7.81 (m, 1H), 7.34−7.31 (m, 1H), 7.22−7.21 (m, 1H), 3.05 (t, *J* = 7.4 Hz, 2H), 2.73 (t, *J* = 7.4 Hz, 2H); ^{13}C{^{1}H} NMR (125 MHz, $CDCl_3$) δ 156.8, 155.7, 149.8, 149.2, 147.7, 137.2, 124.1, 123.6, 121.4, 120.7, 118.6, 31.0, 18.3; HRMS (DART) calcd for $(M+H)^+$ $C_{13}H_{12}N_3$: 210.1025, found 210.1023.

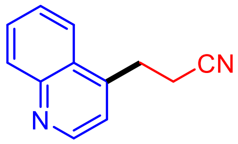

4hA; using $CHCl_3$/MeOH = 1:0 to 19:1 as the eluent, 30.6 mg, 87%, colorless oil; IR (neat, cm^{-1}) 3063, 2950, 2247; ^{1}H NMR (500 MHz, $CDCl_3$) δ 8.87 (d, *J* = 4.3 Hz, 1H), 8.16 (d, *J* = 8.6 Hz, 1H), 7.93 (d, *J* = 8.6 Hz, 1H), 7.76−7.73 (m, 1H), 7.63−7.60 (m, 1H), 7.29 (d, *J* = 4.3 Hz, 1H), 3.42 (t, *J* = 7.4 Hz, 2H), 2.80 (t, *J* = 7.4 Hz, 2H); ^{13}C{^{1}H} NMR (125 MHz, $CDCl_3$) δ 150.3, 148.4, 143.4, 130.7, 129.7, 127.3, 126.6, 122.5, 120.9, 118.6, 27.8, 17.7; HRMS (DART) calcd for $(M+H)^+$ $C_{12}H_{11}N$: 183.0916, found 183.0922.

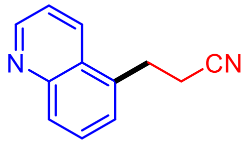

4iA; using $CHCl_3$/MeOH = 1:0 to 19:1 as the eluent, 30.6 mg, 42%, colorless oil; IR (neat, cm^{-1}); 3063, 2950, 2246; ^{1}H NMR (500 MHz, $CDCl_3$) δ 8.97−8.96 (m, 1H), 8.30 (d, *J* = 8.6 Hz, 1H), 8.08 (d, *J* = 8.6 Hz, 1H), 7.71−7.68 (m, 1H), 7.50−7.48 (m, 2H), 3.45 (t, *J* = 7.5 Hz, 2H), 2.77 (t, *J* = 7.5 Hz, 2H); ^{13}C{^{1}H} NMR (125 MHz, $CDCl_3$) δ 150.4, 148.9, 134.3, 131.2, 129.7, 129.3, 127.1, 126.4, 121.5, 118.9, 28.0, 18.8; HRMS (DART) calcd for $(M+H)^+$ $C_{12}H_{11}N$: 183.0916, found 183.0909.

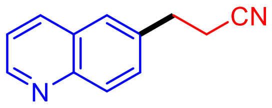

4jA; using $CHCl_3$/MeOH = 1:0 to 19:1 as the eluent, 52.5 mg, 72%, colorless oil; IR (neat, cm^{-1});

3034, 2932, 2246; ^{1}H NMR (500 MHz, $CDCl_3$) δ 8.91−8.90 (m, 1H), 8.14−8.09 (m, 2H), 7.69 (d, *J* = 1.1 Hz, 1H), 7.59−7.57 (m, 1H), 7.43−7.40 (m, 1H), 3.15 (t, *J* = 7.4 Hz, 2H), 2.74 (t, *J* = 7.4 Hz, 2H); $^{13}C\{^1H\}$ NMR (125 MHz, $CDCl_3$) δ 150.6, 147.6, 136.4, 135.9, 130.3, 130.0, 128.4, 126.9, 121.6, 119.0, 31.6, 19.3; HRMS (DART) calcd for $(M+H)^+$ $C_{12}H_{11}N$: 183.0916, found 183.0911.

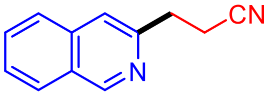

4kA; using $CHCl_3$/MeOH = 1:0 to 19:1 as the eluent, 59.0 mg, 81%, white solid, m.p. 55−56 °C; IR (KBr, cm^{-1}) 2959, 2928, 2243; ^{1}H NMR (500 MHz, $CDCl_3$) δ 9.23 (s, 1H), 7.97 (d, *J* = 8.0 Hz, 1H), 7.80 (d, *J* = 8.0 Hz, 1H), 7.72−7.69 (m, 1H), 7.61−7.58 (m, 2H), 3.26 (t, *J* = 7.4 Hz, 2H), 2.93 (t, *J* = 7.4 Hz, 2H); $^{13}C\{^1H\}$ NMR (125 MHz, $CDCl_3$) δ 152.8, 150.6, 136.4, 130.8, 127.7, 127.7, 127.3, 126.5, 119.6, 119.2, 33.6, 17.5; HRMS (DART) calcd for $(M+H)^+$ $C_{12}H_{11}N$: 183.0916, found 183.0910.

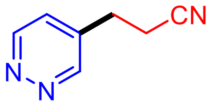

4lA; using $CHCl_3$/MeOH = 1:0 to 19:1 as the eluent, 40.5 mg, 76%, colorless oil; IR (neat, cm^{-1}) 3052, 2934, 2247; ^{1}H NMR (500 MHz, $CDCl_3$) δ 9.18 (d, *J* = 5.2 Hz, 1H), 9.15 (s, 1H), 7.45−7.43 (m, 1H), 3.03 (t, *J* = 7.2 Hz, 2H), 2.75 (t, *J* = 7.2 Hz, 2H); $^{13}C\{^1H\}$ NMR (125 MHz, $CDCl_3$) δ 152.1, 151.4, 137.2, 126.0, 117.9, 28.3, 17.8; HRMS (DART) calcd for $(M+H)^+$ $C_7H_8N_3$: 134.0712, found 134.0717.

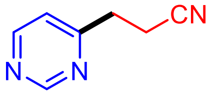

4mA; using $CHCl_3$/MeOH = 1:0 to 19:1 as the eluent, 28.8 mg, 54%, colorless oil; IR (neat, cm^{-1}); 2931, 2855, 2247; ^{1}H NMR (500 MHz, $CDCl_3$) δ 9.19 (s, 1H), 8.69 (d, *J* = 4.6 Hz, 1H), 7.27 (d, *J* = 4.6 Hz, 1H), 3.13 (t, *J* = 7.2 Hz, 2H), 2.90 (t, *J* = 7.2 Hz, 2H); $^{13}C\{^1H\}$ NMR (125 MHz, $CDCl_3$) δ 165.5, 159.1, 157.3, 120.8, 118.9, 32.7, 15.5; HRMS (DART) calcd for $(M+H)^+$ $C_7H_8N_3$: 134.0712, found 134.0721.

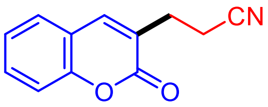

4nA; using hexane/$CHCl_3$ = 2:1 as the eluent, 5.6 mg, 7%, white solid, m.p. 96−97 °C; IR (KBr, cm^{-1}); 2998, 2938, 2239, 1712; ^{1}H NMR (500 MHz, $CDCl_3$); δ 7.71 (s, 1H), 7.56−7.50 (m, 2H), 7.36−7.29

(m, 2H), 2.91 (t, J = 6.6 Hz, 2H), 2.81 (t, J = 6.6 Hz, 2H) $^{13}C\{^1H\}$ NMR (125 MHz, $CDCl_3$) δ 161.3, 153.6, 141.6, 131.8, 127.9, 125.1, 124.8, 119.0, 118.9, 116.7, 27.9, 16.4; HRMS (DART) calcd for $(M+H)^+$ $C_{12}H_{10}NO_2$: 200.0706, found 200.0704.

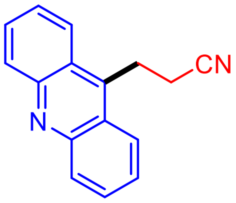

4oA; using $CHCl_3$/MeOH = 1:0 to 19:1 as the eluent, 12.1 mg, 13%, yellow solid, m.p. 178−179 °C; IR (KBr, cm^{-1}); 2921, 2857, 2243; ^1H NMR (500 MHz, $CDCl_3$); δ 8.28 (d, J = 8.6 Hz, 2H), 8.20 (d, J = 8.6 Hz, 2H), 7.84−7.81 (m, 2H), 7.67−7.64 (m, 2H), 4.05 (t, J = 8.0 Hz, 2H), 2.85 (t, J = 8.0 Hz, 2H) $^{13}C\{^1H\}$ NMR (125 MHz, $CDCl_3$) δ 148.8, 140.8, 131.0, 130.2, 126.9, 123.1, 118.6, 77.4, 77.1, 76.9, 23.3, 18.2; HRMS (DART) calcd for $(M+H)^+$ $C_{16}H_{13}N_2$: 233.1073, found 233.1074.

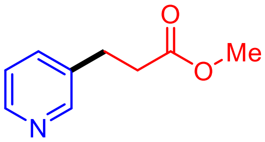

4eB; Compound **4eB** has been previously reported.[S4] using $CHCl_3$/MeOH = 1:0 to 19:1 as the eluent, 25.8 mg, 39%, colorless oil, ^1H NMR (500 MHz, $CDCl_3$) δ 8.47 (t, J = 4.9 Hz, 2H), 7.53 (d, J = 7.4 Hz, 1H), 7.22 (dd, J = 7.4, 4.9 Hz, 1H), 3.67 (s, 3H), 2.96 (t, J = 7.7 Hz, 2H), 2.65 (t, J = 7.7 Hz, 2H); $^{13}C\{^1H\}$ NMR (125 MHz, $CDCl_3$) δ 172.9, 149.9, 147.9, 135.9, 123.5, 51.8, 35.2, 28.1.

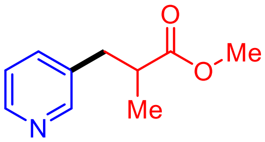

4eC; using $CHCl_3$/MeOH = 1:0 to 19:1 as the eluent, 49.5 mg, 69%, colorless oil, IR (neat, cm^{-1}); 2924, 2852, 1738; ^1H NMR (500 MHz, $CDCl_3$) δ 8.47−8.44 (m, 2H), 7.49 (d, J = 8.0 Hz, 1H), 7.23−7.21 (m, 1H), 3.64 (s, 2H), 3.03−2.99 (m, 1H), 2.77−2.68 (m, 2H), 1.19 (d, J = 6.9 Hz, 3H); $^{13}C\{^1H\}$ NMR (125 MHz, $CDCl_3$) δ 176.0, 150.4, 148.0, 136.5, 134.8, 123.4, 51.8, 41.2, 36.8, 16.9; HRMS (DART) calcd for $(M+H)^+$ $C_{10}H_{14}NO_2$: 180.1019, found 180.1010.

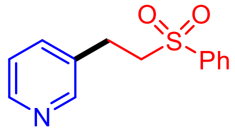

4eD; using $CHCl_3$/MeOH = 1:0 to 10:1 as the eluent, 75.2 mg, 76%, colorless oil, IR (neat, cm^{-1}); 2924, 2853, 1576; ^1H NMR (500 MHz, $CDCl_3$) δ 8.44 (d, J = 3.4 Hz, 1H), 8.38 (s, 1H), 7.91 (d, J = 7.4 Hz, 2H), 7.67−7.64 (m, 1H), 7.56 (t, J = 7.7 Hz, 2H), 7.45 (d, J = 8.0 Hz, 1H), 7.18 (dd, J = 7.7, 4.9 Hz, 1H), 3.36−3.33 (m, 2H), 3.08−3.04 (m, 2H); $^{13}C\{^1H\}$ NMR (125 MHz, $CDCl_3$) δ 149.7, 148.5, 138.8, 135.9, 134.1, 133.1, 129.5, 128.1, 123.7, 56.9, 26.1; HRMS (DART) calcd for $(M+H)^+$ $C_{13}H_{14}NO_2S$: 248.0739, found 248.0742.

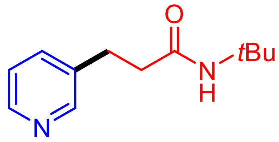

4eE; using $CHCl_3$/MeOH = 1:0 to 19:1 as the eluent, 26.4 mg, 32%, colorless oil, IR (neat, cm^{-1}); 3298, 2923, 2853, 1648; ^1H NMR (500 MHz, $CDCl_3$) δ 8.44−8.42 (m, 2H), 7.52 (d, J = 8.0 Hz, 1H), 7.18 (d, J = 8.0 Hz, 1H), 5.21 (s, NH), 2.93 (t, J = 7.4 Hz, 2H), 2.36 (t, J = 7.4 Hz, 2H), 1.27 (s, 9H); $^{13}C\{^1H\}$ NMR (125 MHz, $CDCl_3$) δ 170.7, 149.9, 147.8, 136.5, 136.2, 123.4, 51.4, 38.9, 29.8, 28.8; HRMS (DART) calcd for $(M+H)^+$ $C_{12}H_{19}N_2O$: 207.1491, found 207.1489.

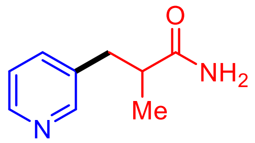

4eF; using $CHCl_3$/MeOH = 1:0 to 10:1 as the eluent, 19.7 mg, 30%, colorless oil, IR (neat, cm^{-1});3382, 2923, 2853, 1668; ^1H NMR (500 MHz, $CDCl_3$) δ 8.35−8.32 (m, 2H), 7.46 (d, J = 7.4 Hz, 1H), 7.14 (dd, J = 7.7, 4.9 Hz, 1H), 6.26 (s, 1H), 6.10 (s, 1H), 2.94−2.90 (m, 1H), 2.61−2.49 (m, 2H), 1.13 (d, J = 6.9 Hz, 3H); $^{13}C\{^1H\}$ NMR (125 MHz, $CDCl_3$) δ 178.2, 150.1, 147.6, 136.8, 135.4, 123.5, 42.5, 37.2, 17.8; HRMS (DART) calcd for $(M+H)^+$ $C_9H_{13}N_2O$: 165.1022, found 165.1018.

References in SI

(S1) S. Kubosaki, H. Takeuchi, Y. Iwata, Y. Tanaka, M. Yamawaki, T. Morita, Y. Yoshimi, Visible- and UV- Light-Induced Decarboxylative Radical Reactions of Benzoic Acid Using Organic Photoredox Catalysts, *J. Org. Chem.*, **2020**, *85*, 5362−5369.

(S2) B. Anxionnat, G. D. Pardo, G. Ricci, J. Cossy, Monoalkylation of Acetonitrile by Primary Alcohols Catalyzed by Iridium Complexes, *Org. Lett*, **2011**, *13*, 4084−4087.

(S3) S. Zhang, L. Li, X. Li, J. Zhang, G. Li, M. Findlater, Electroreductive 4-Pyridylation of Electron-deficient Alkenes with Assistance of $Ni(acac)_2$, *Org. Lett.*, **2020**, *22*, 3570−3575.
(S4) K. Geoghegan, S. Kelleher, P. Evans, An Investigation into the One-Pot Heck Olefination-Hydrogeneration Reaction, *J. Org. Chem.* **2011**, *76*, 2187−2194.

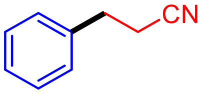

4aA

^{1}H NMR (500 MHz, $CDCl_3$)

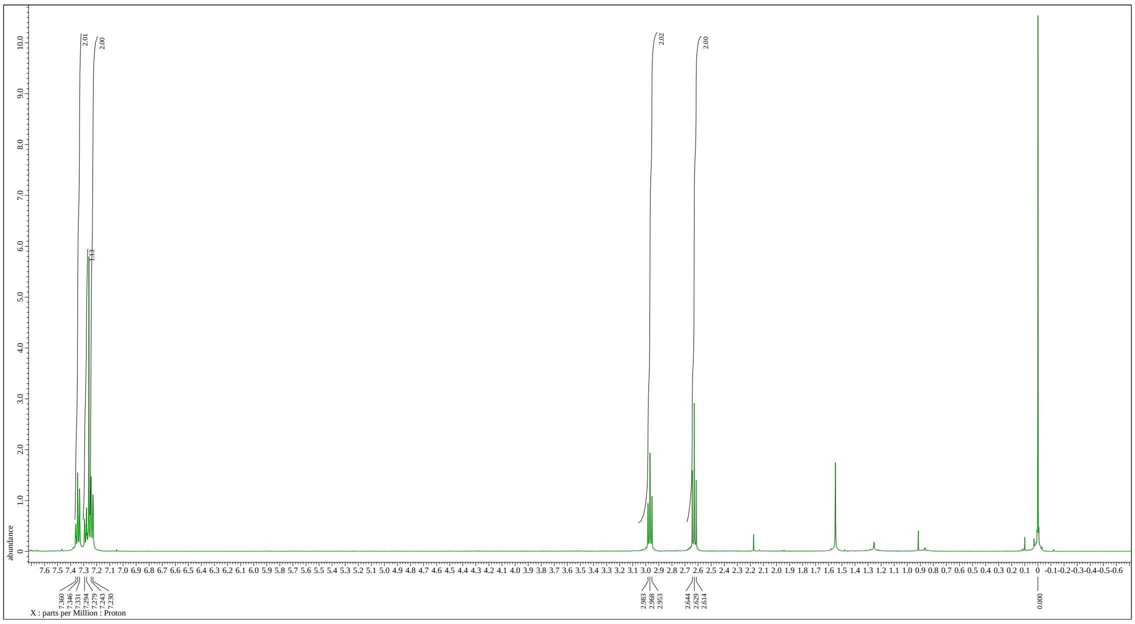

$^{13}C\{^{1}H\}$ NMR (125 MHz, $CDCl_3$)

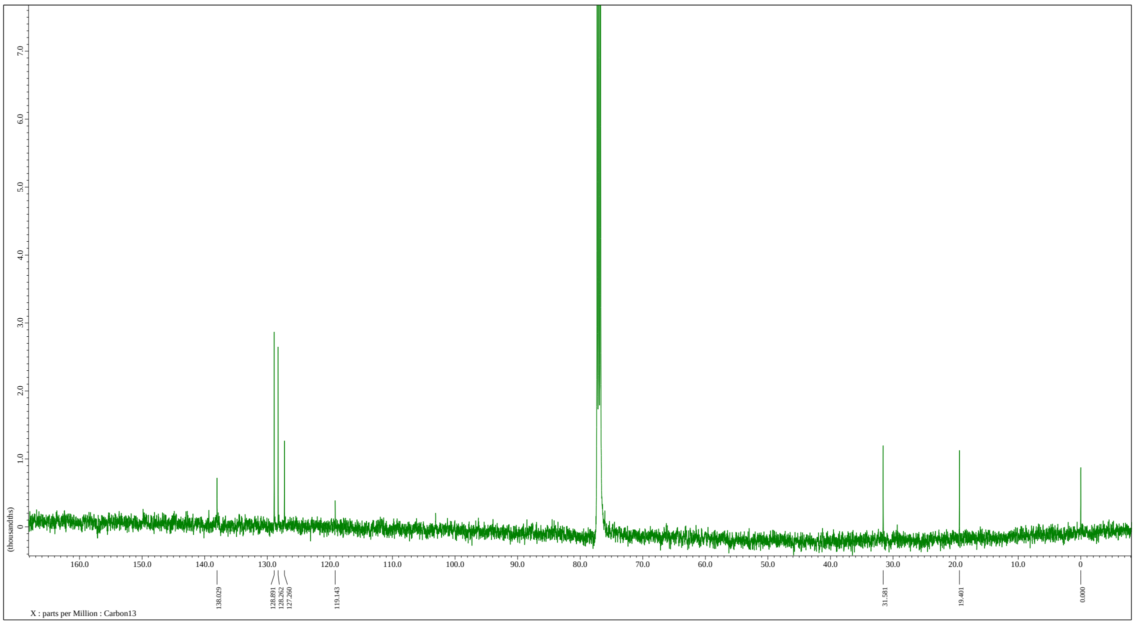

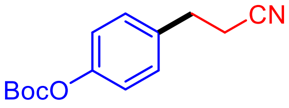

4bA

^{1}H NMR (500 MHz, $CDCl_3$)

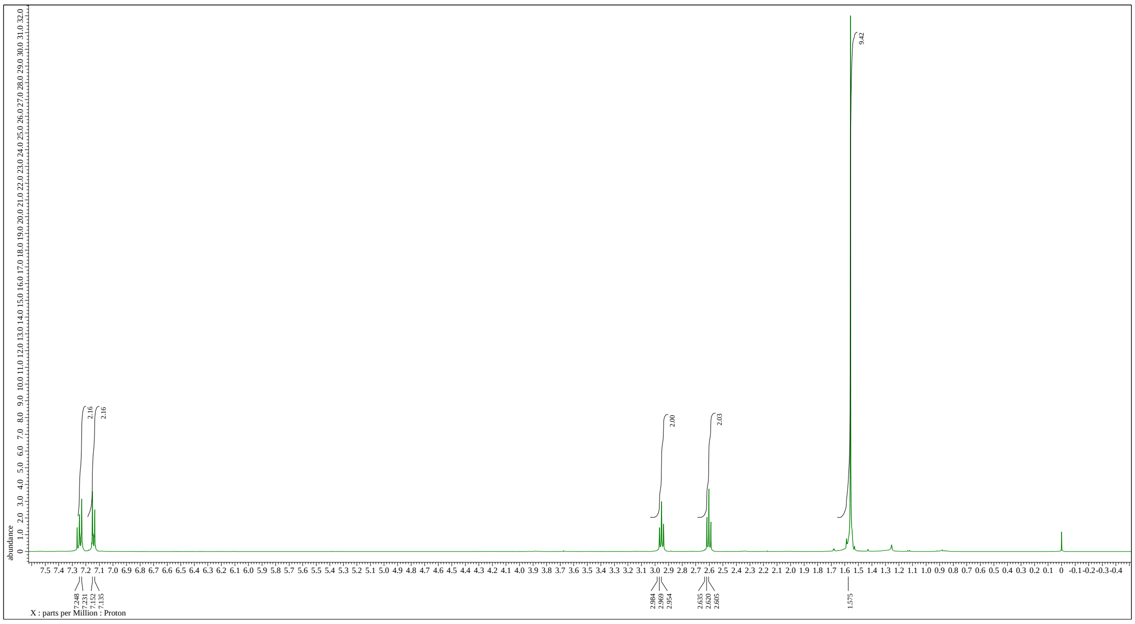

^{13}C{^{1}H} NMR (125 MHz, $CDCl_3$)

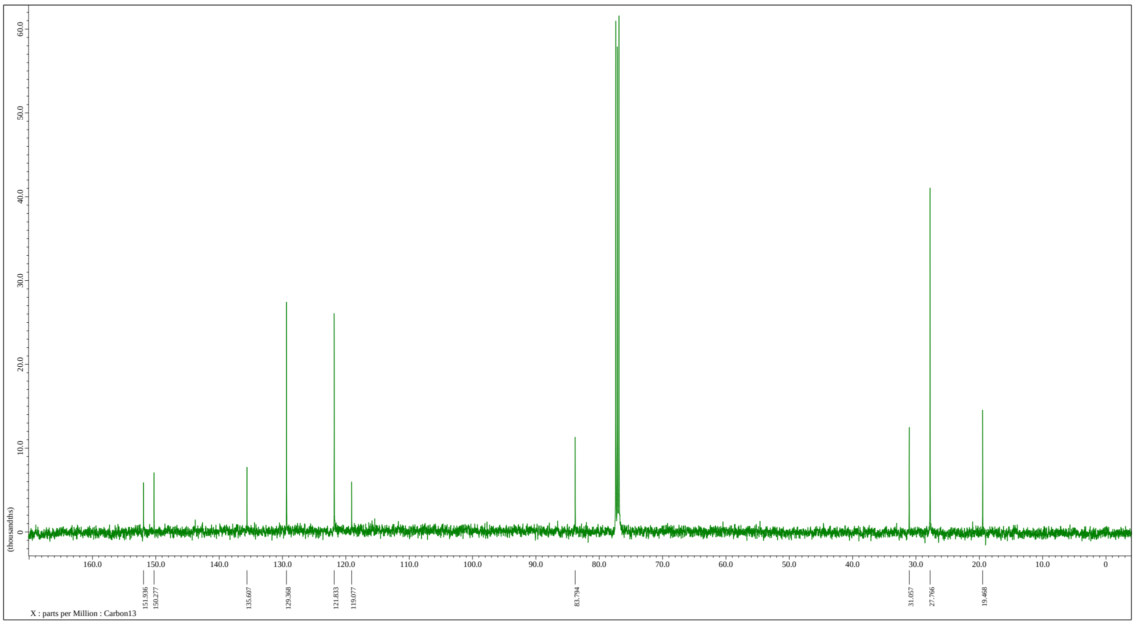

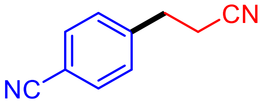

4cA

^{1}H NMR (500 MHz, $CDCl_3$)

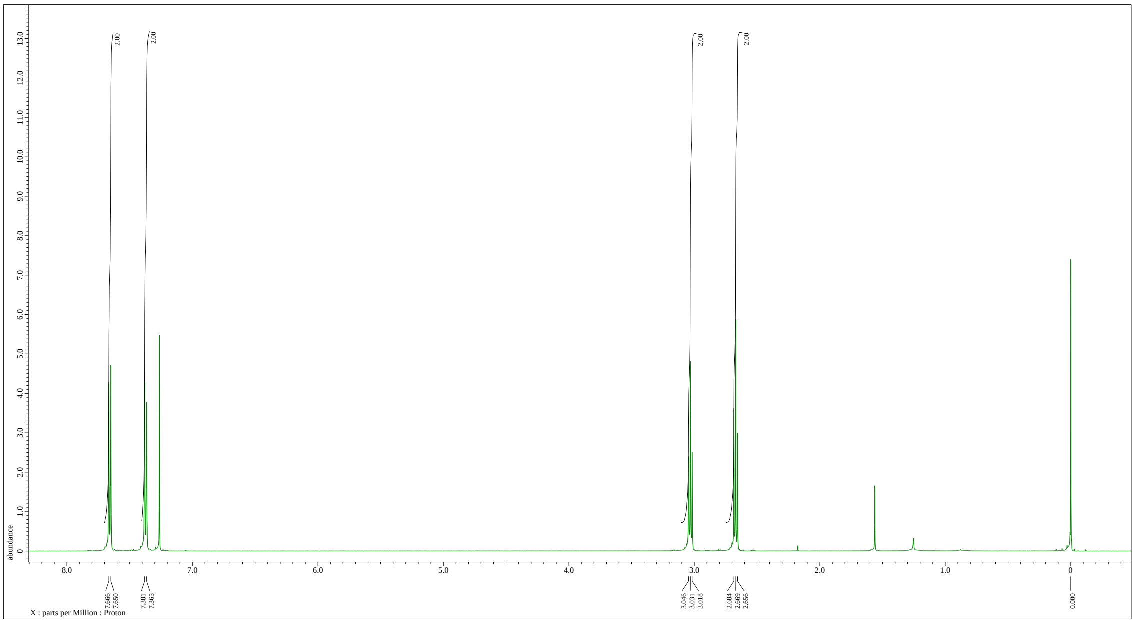

^{13}C{^{1}H} NMR (125 MHz, $CDCl_3$)

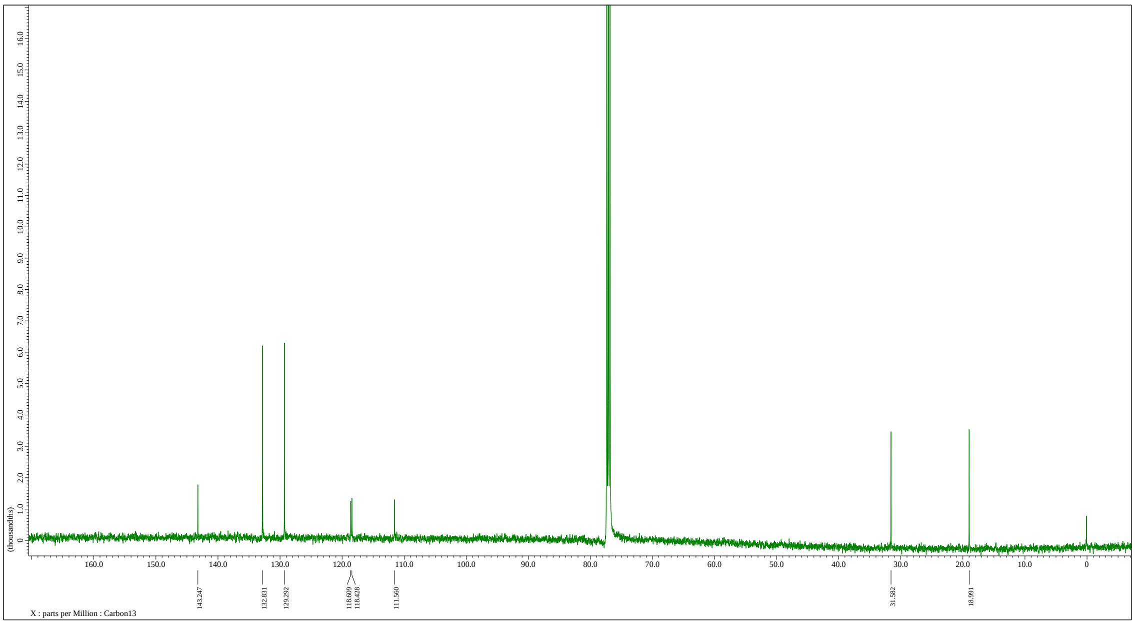

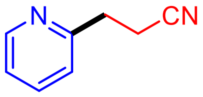

4dA

^{1}H NMR (500 MHz, $CDCl_3$)

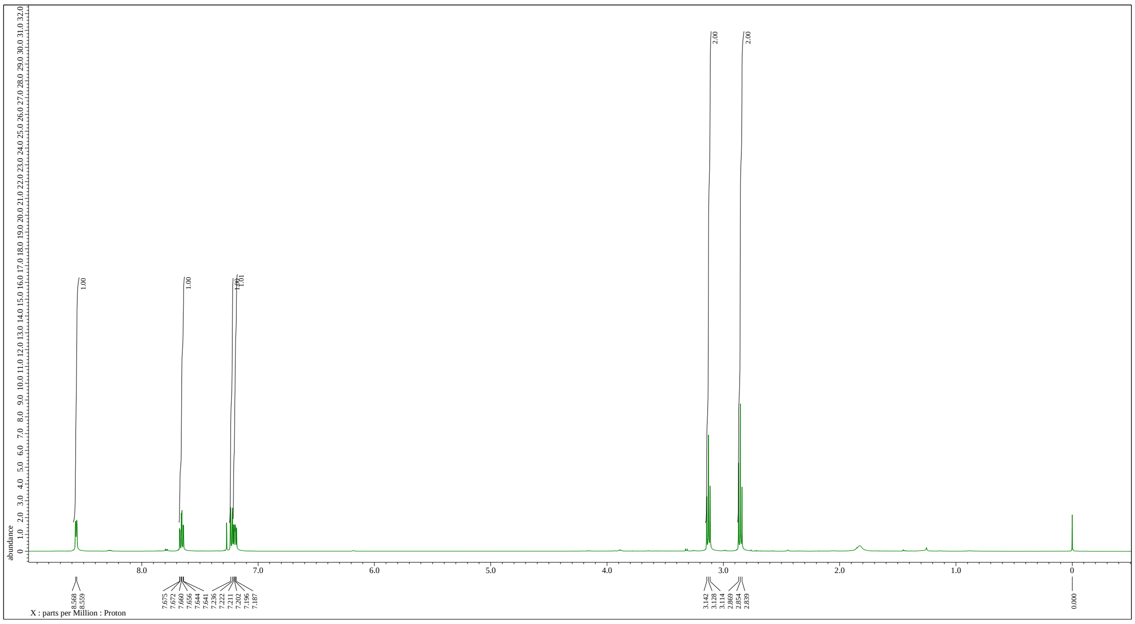

$^{13}C\{^{1}H\}$ NMR (125 MHz, $CDCl_3$)

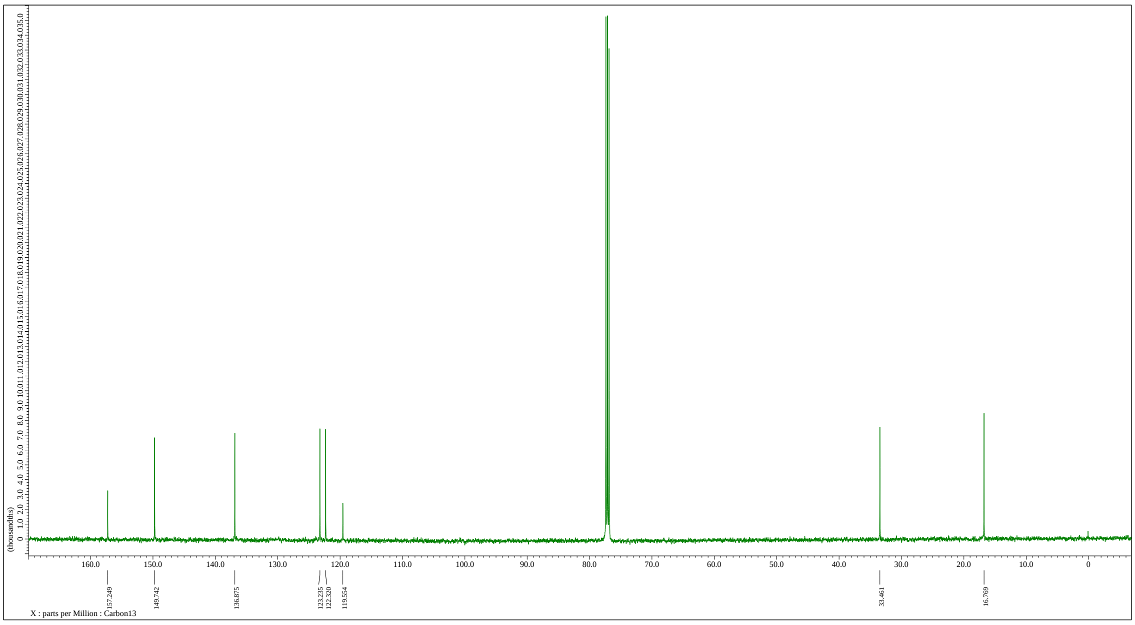

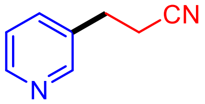

4eA

^{1}H NMR (500 MHz, $CDCl_3$)

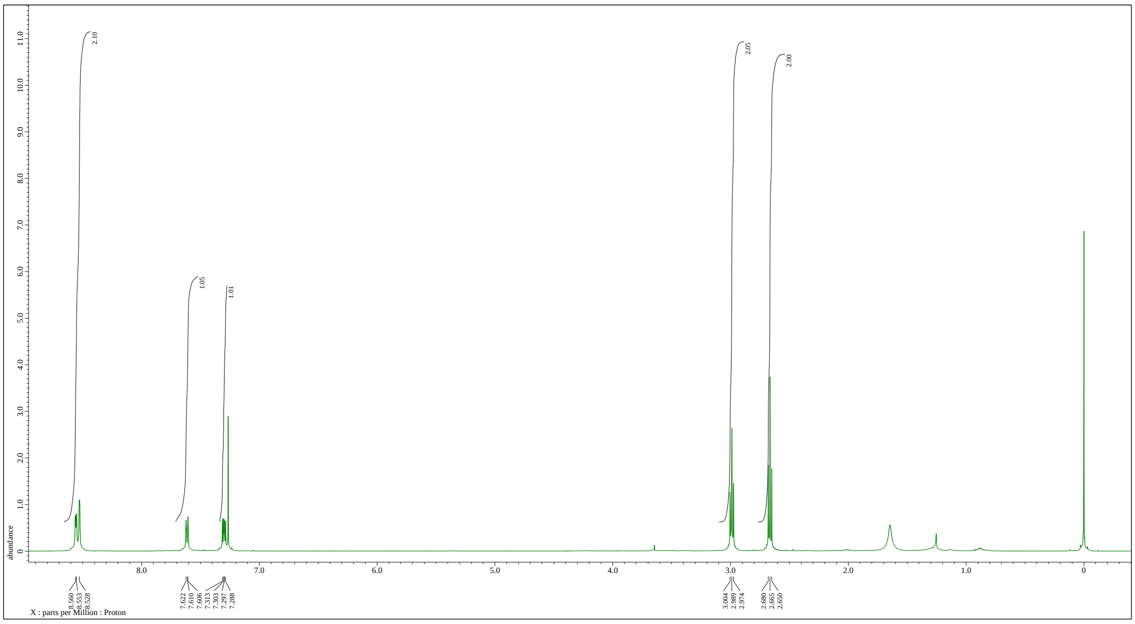

^{13}C{^{1}H} NMR (125 MHz, $CDCl_3$)

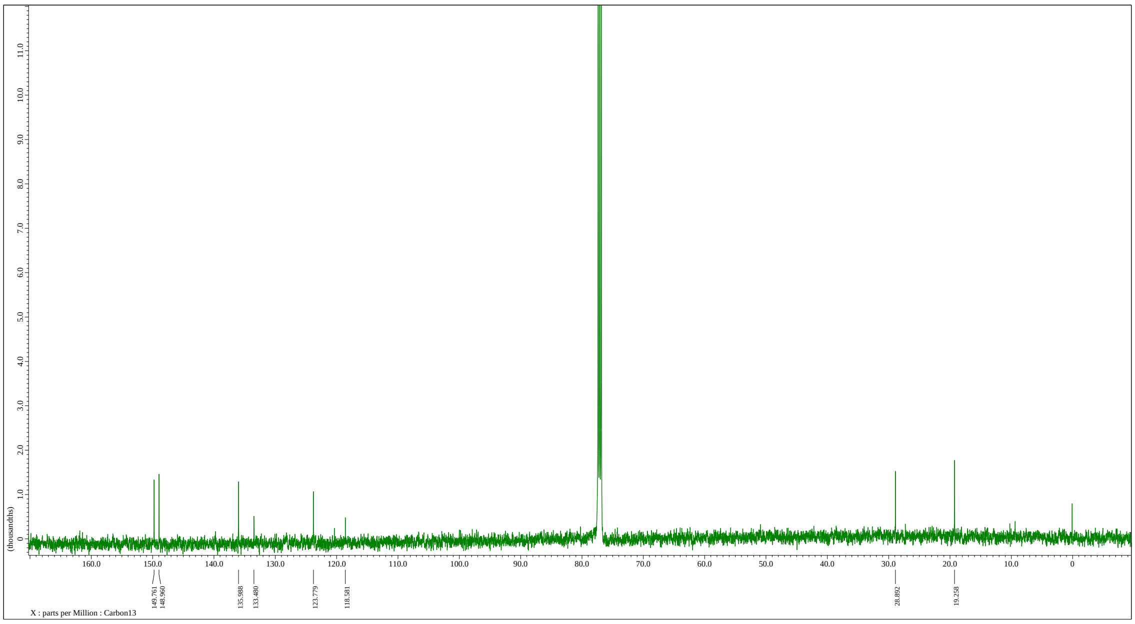

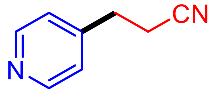

4fA

^{1}H NMR (500 MHz, $CDCl_3$)

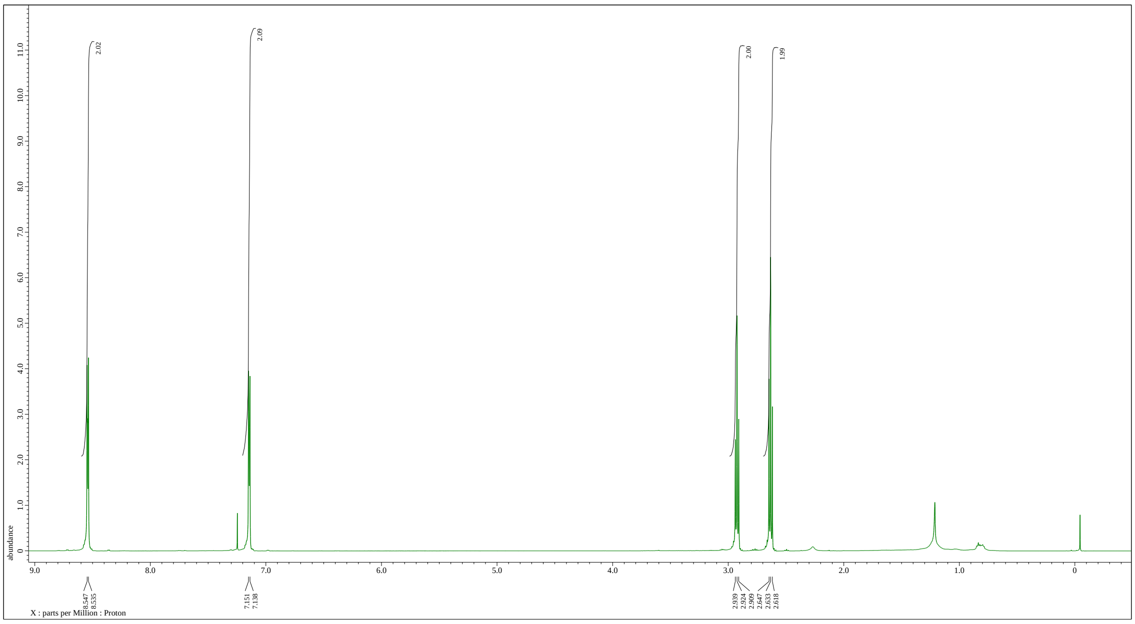

$^{13}C\{^{1}H\}$ NMR (125 MHz, $CDCl_3$)

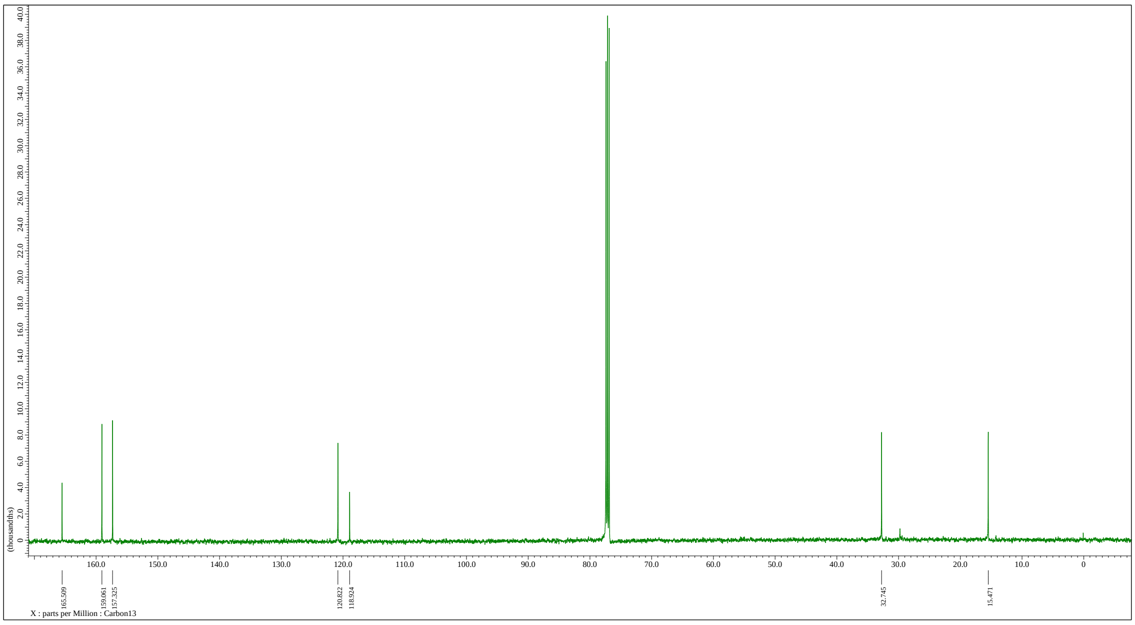

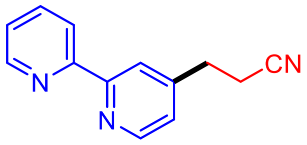

4gA

^{1}H NMR (500 MHz, $CDCl_3$)

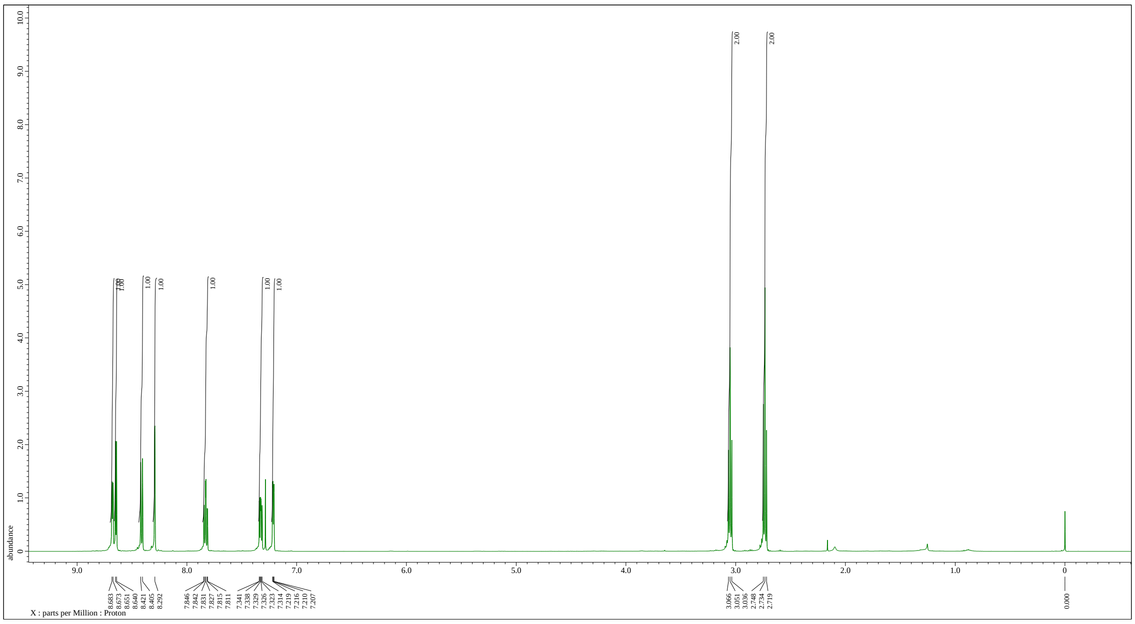

$^{13}C\{^{1}H\}$ NMR (125 MHz, $CDCl_3$)

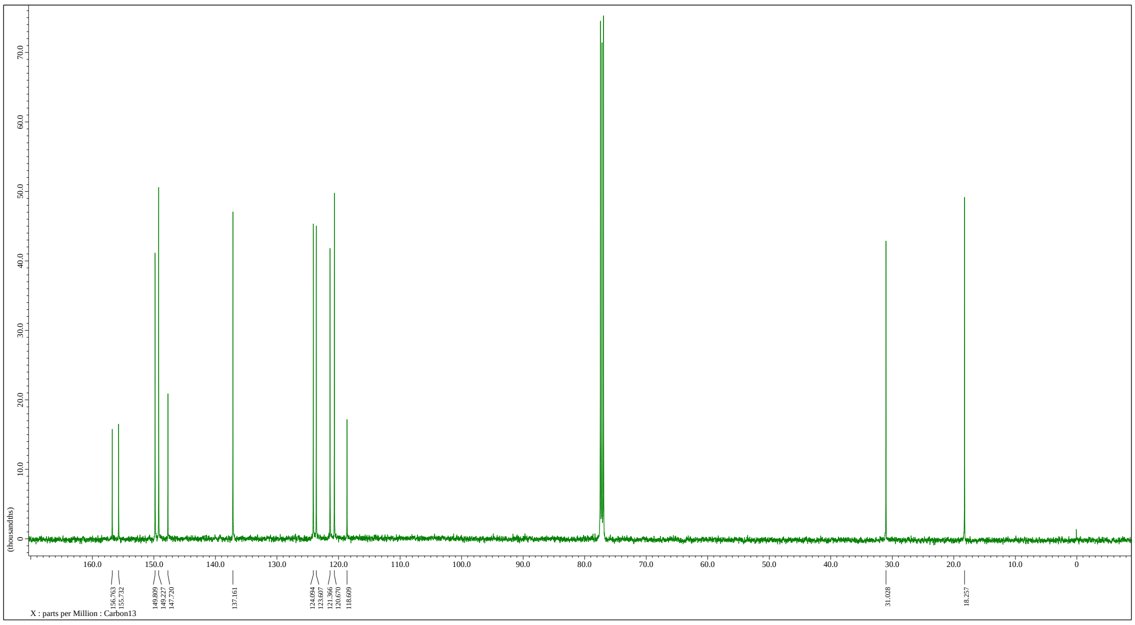

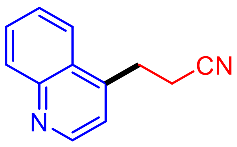

4hA

^{1}H NMR (500 MHz, $CDCl_3$)

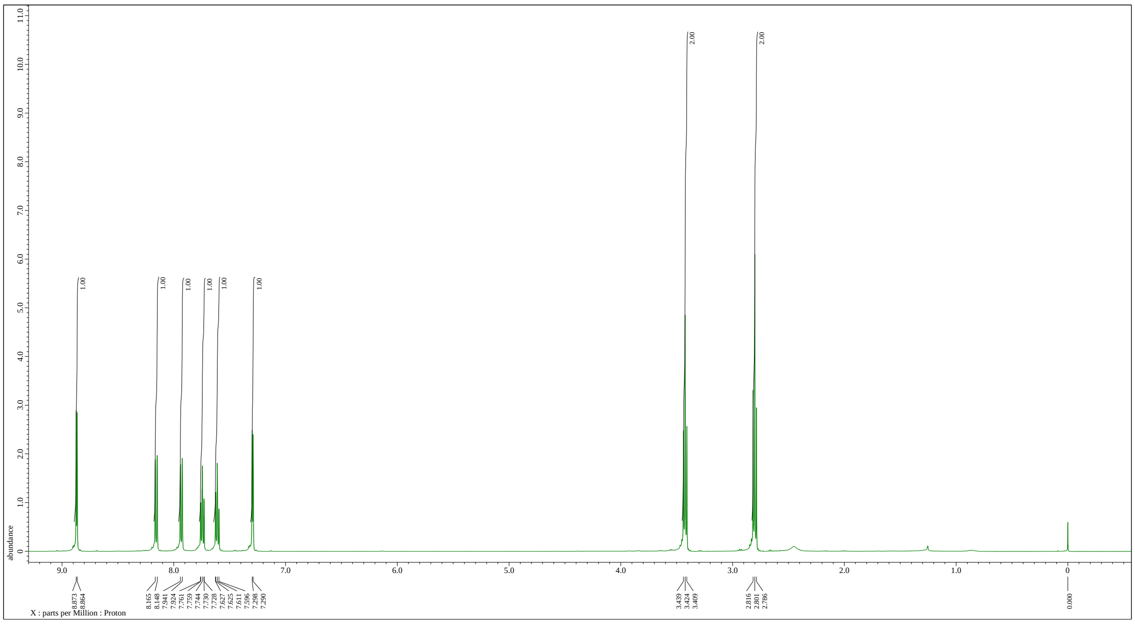

^{13}C{^{1}H} NMR (125 MHz, $CDCl_3$)

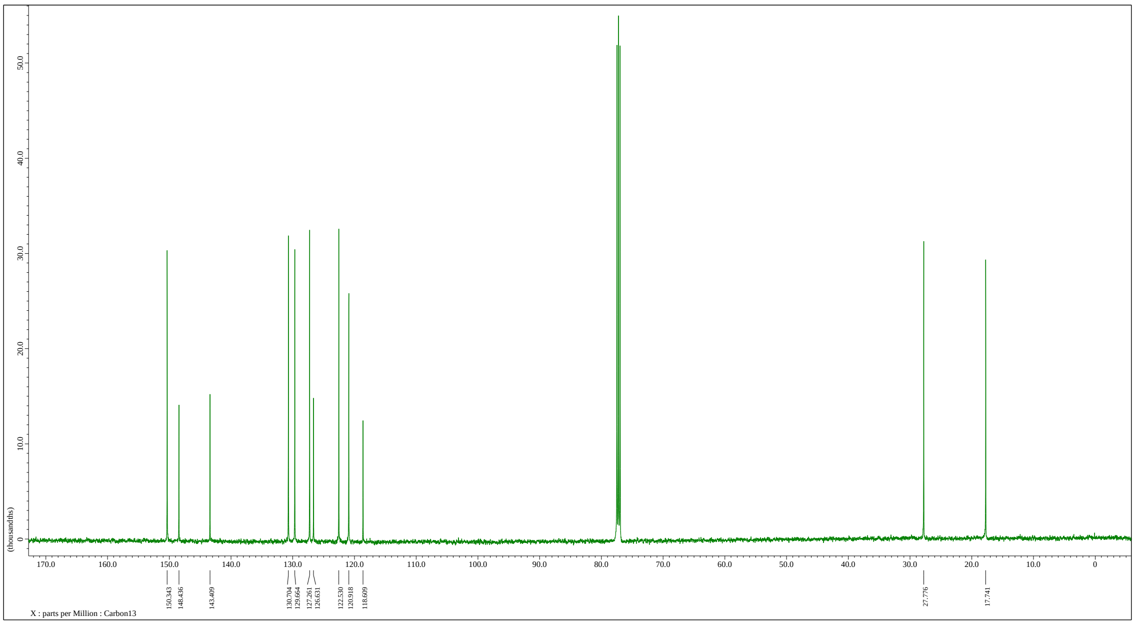

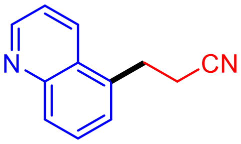

4iA

^{1}H NMR (500 MHz, $CDCl_3$)

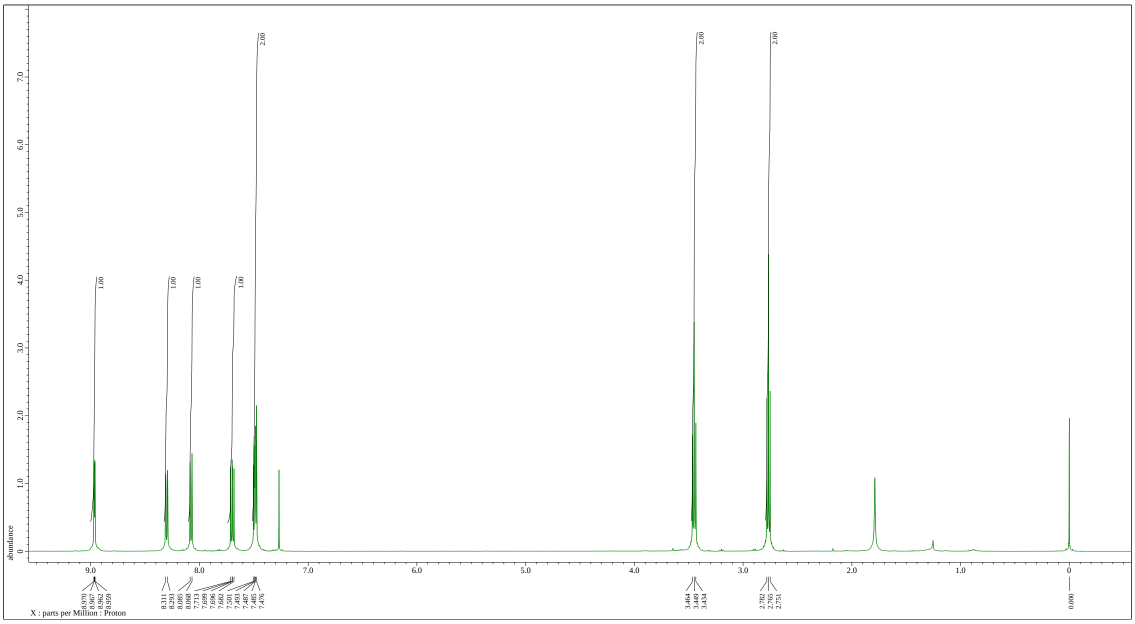

$^{13}C\{^1H\}$ NMR (125 MHz, $CDCl_3$)

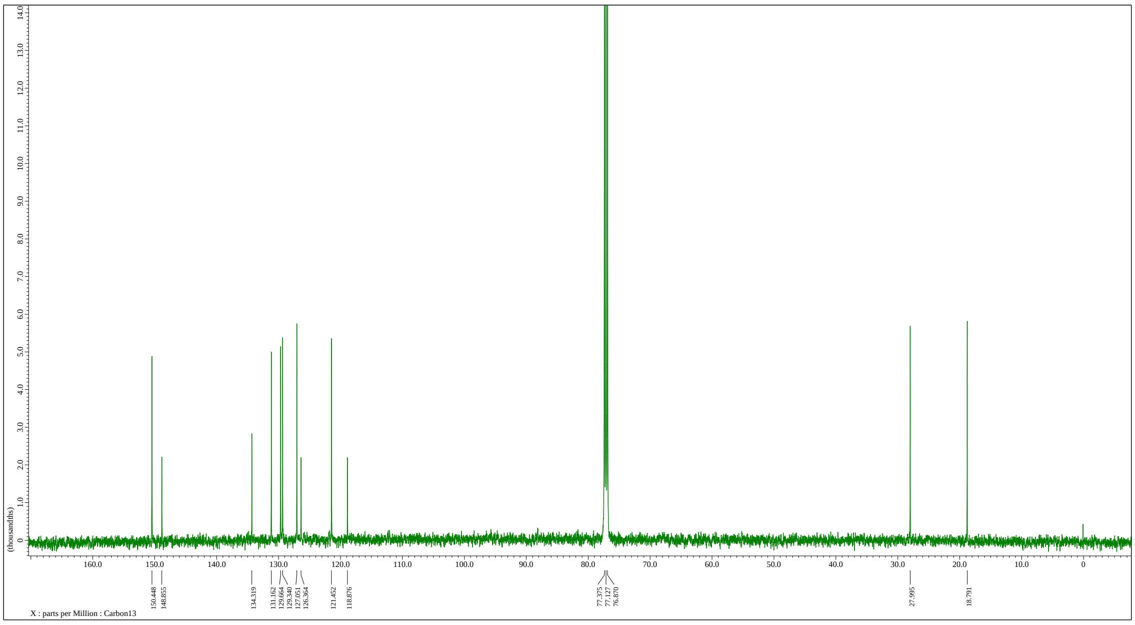

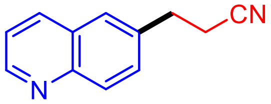

4jA

^{1}H NMR (500 MHz, $CDCl_3$)

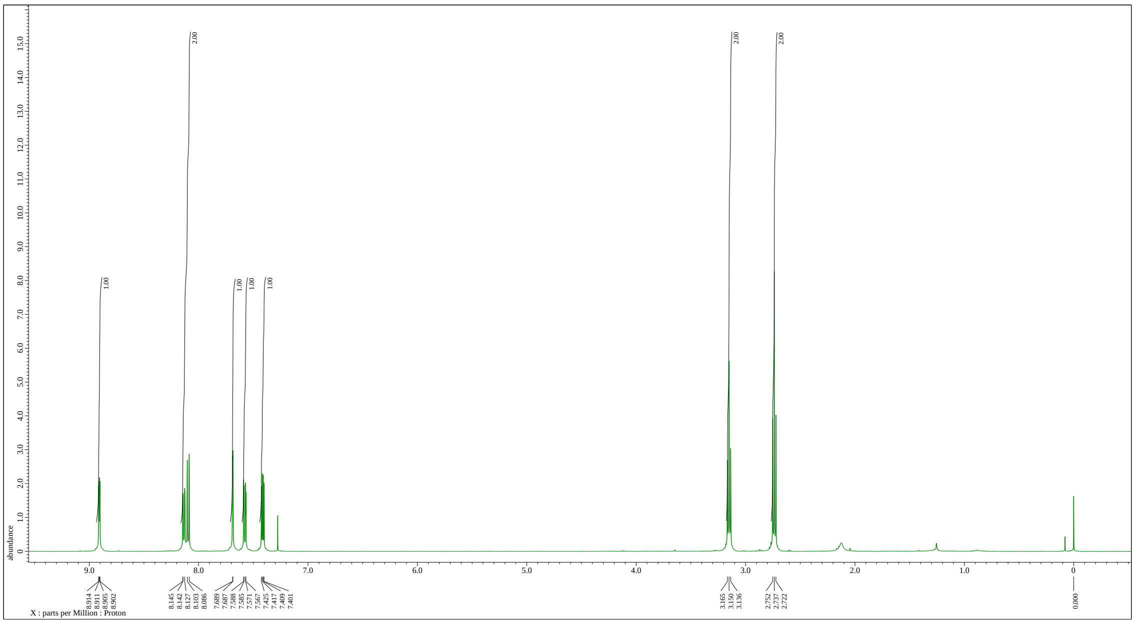

^{13}C{^{1}H} NMR (125 MHz, $CDCl_3$)

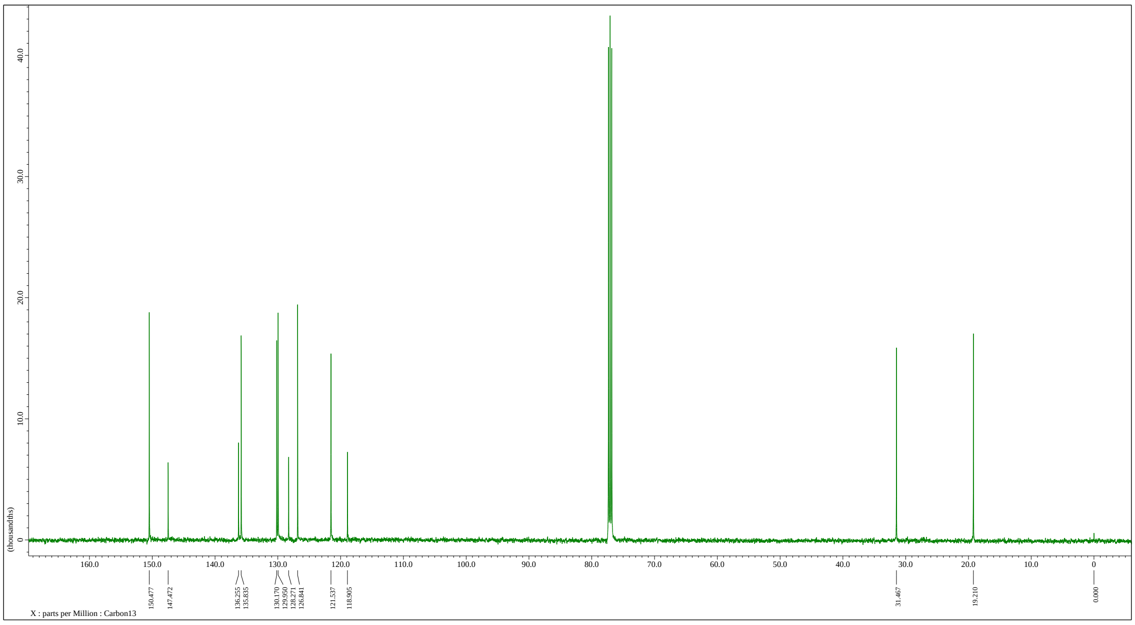

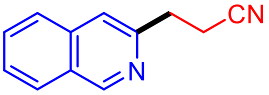

4kA

^{1}H NMR (500 MHz, $CDCl_3$)

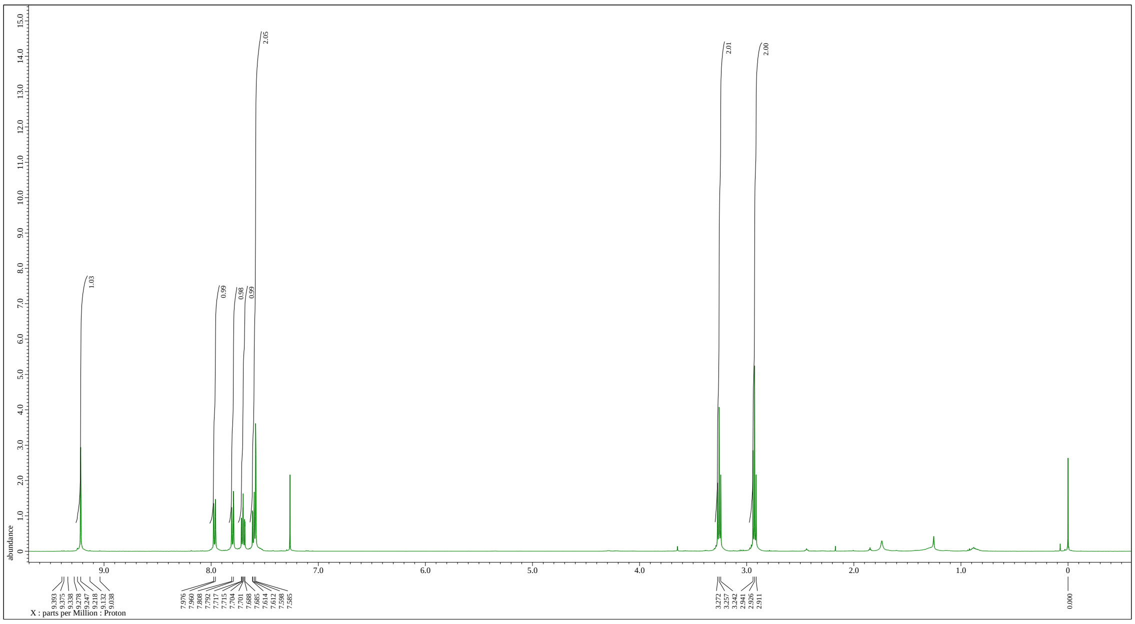

^{13}C{^{1}H} NMR (125 MHz, $CDCl_3$)

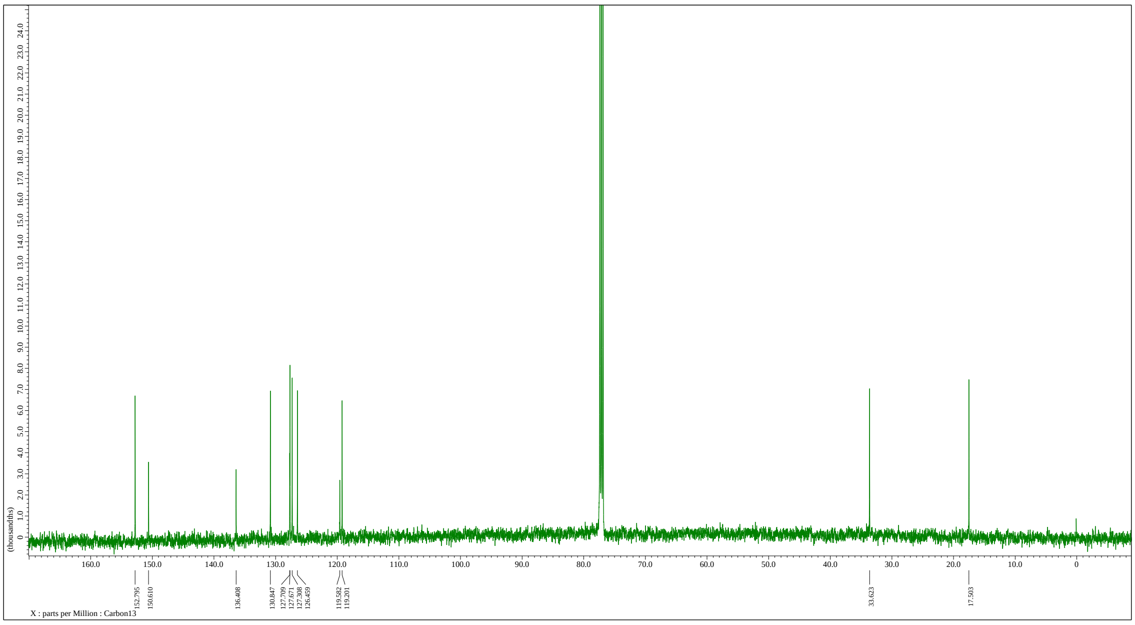

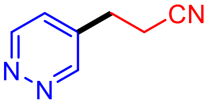

4lA

^{1}H NMR (500 MHz, $CDCl_3$)

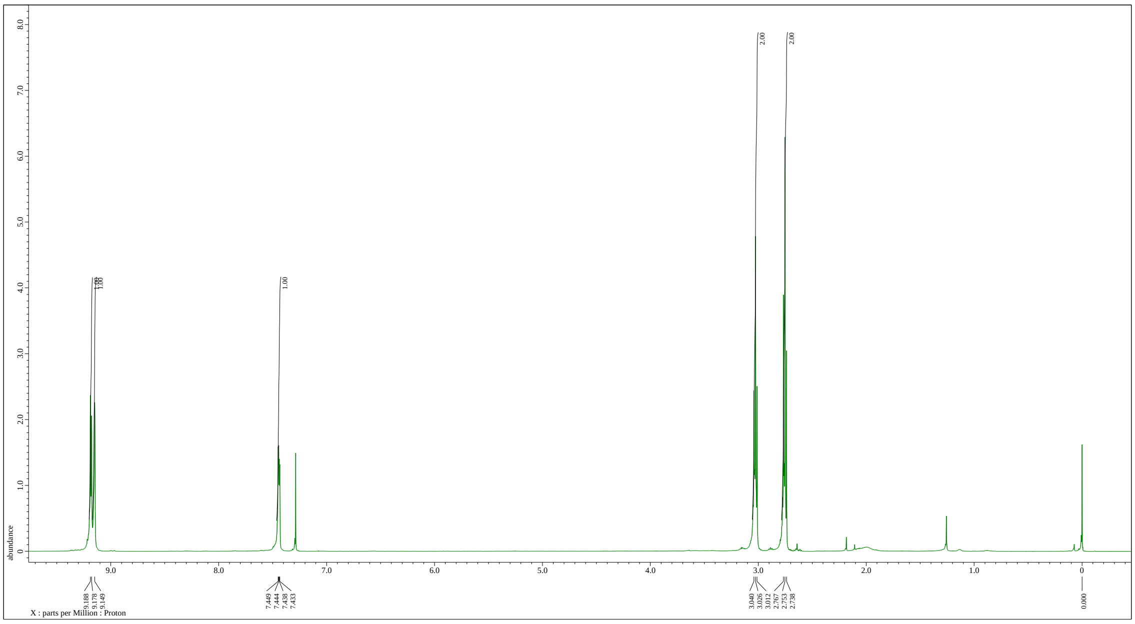

^{13}C{^{1}H} NMR (125 MHz, $CDCl_3$)

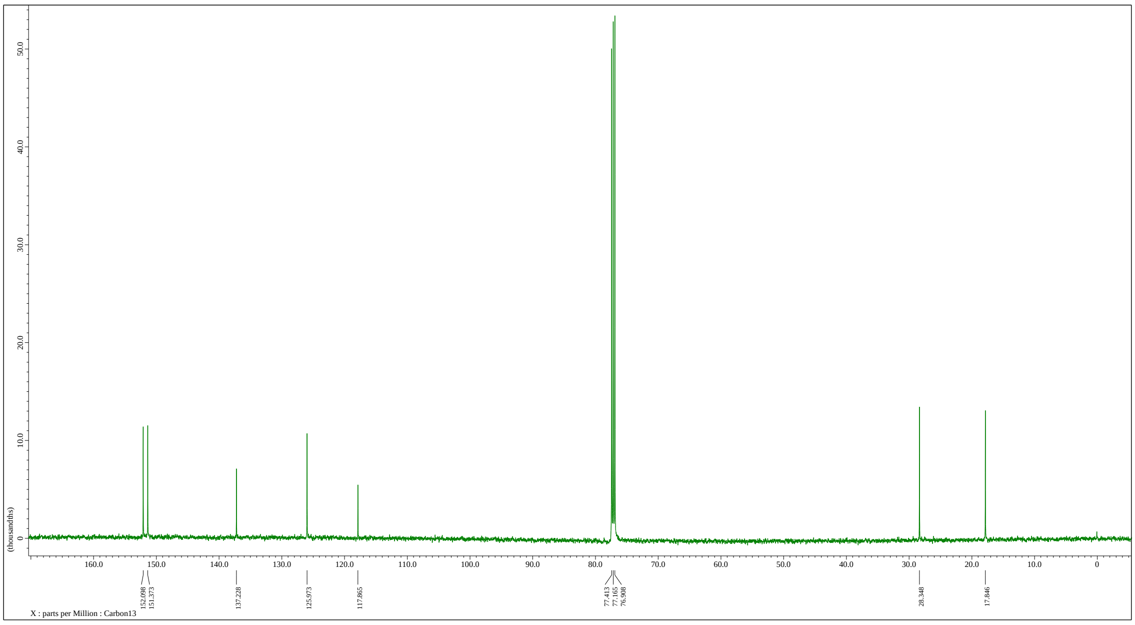

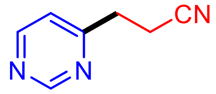

4mA

^{1}H NMR (500 MHz, $CDCl_3$)

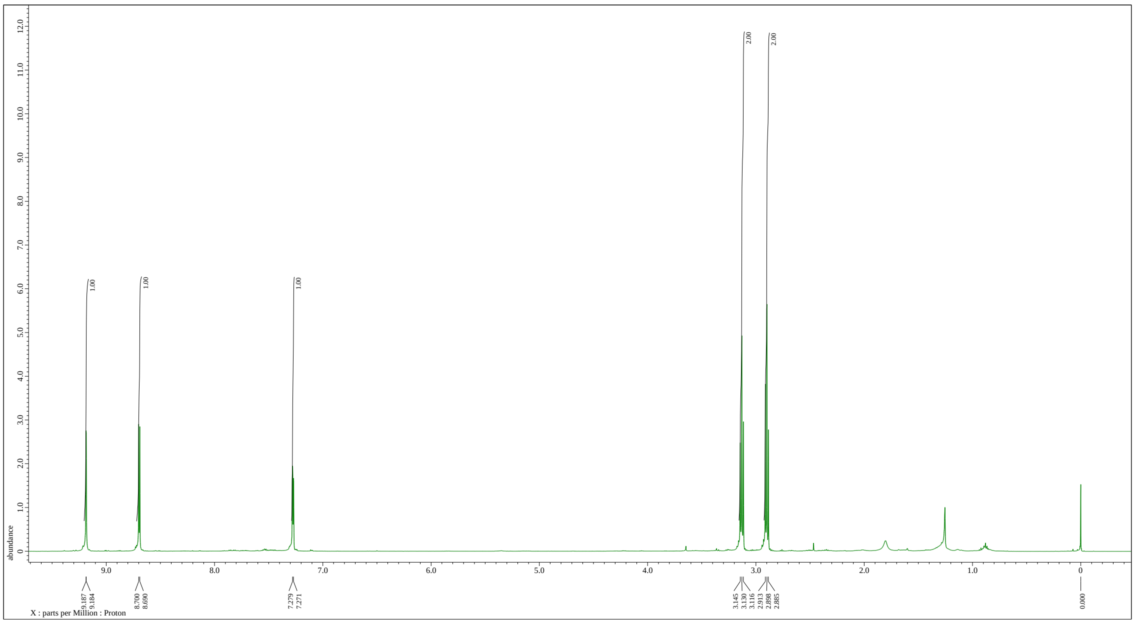

^{13}C{^{1}H} NMR (125 MHz, $CDCl_3$)

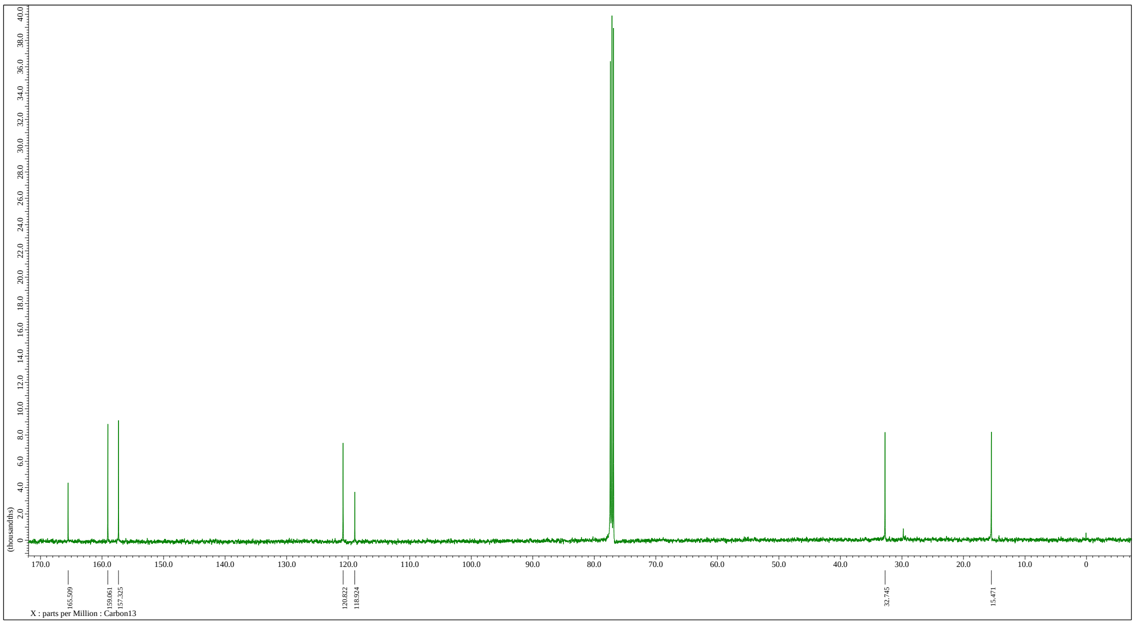

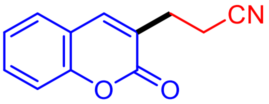

4nA

^{1}H NMR (500 MHz, $CDCl_3$)

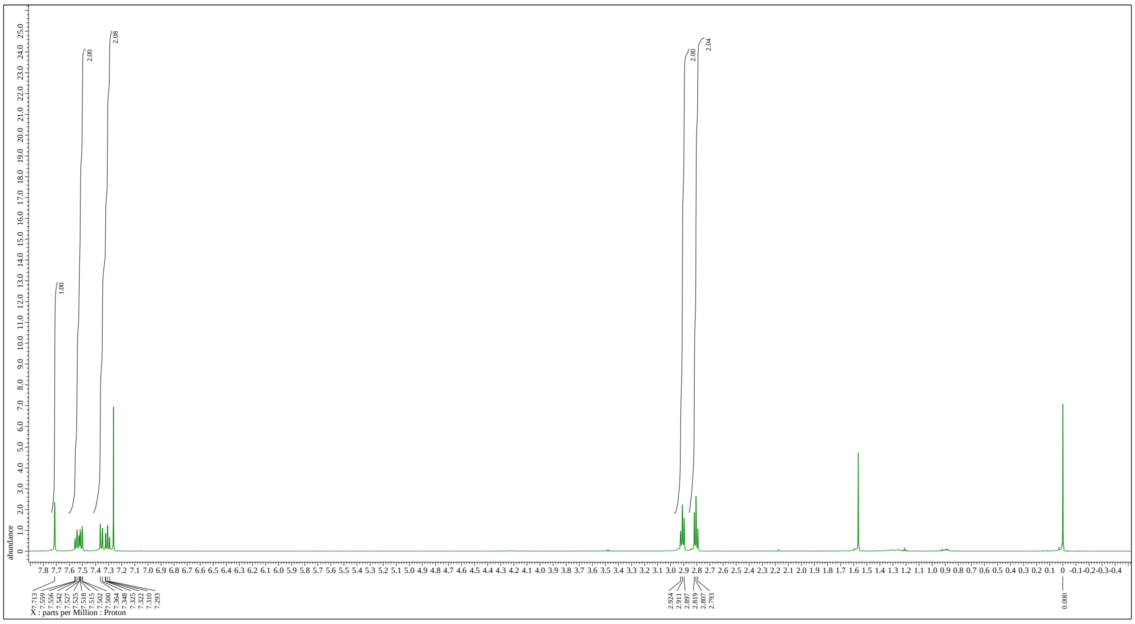

^{13}C{^{1}H} NMR (125 MHz, $CDCl_3$)

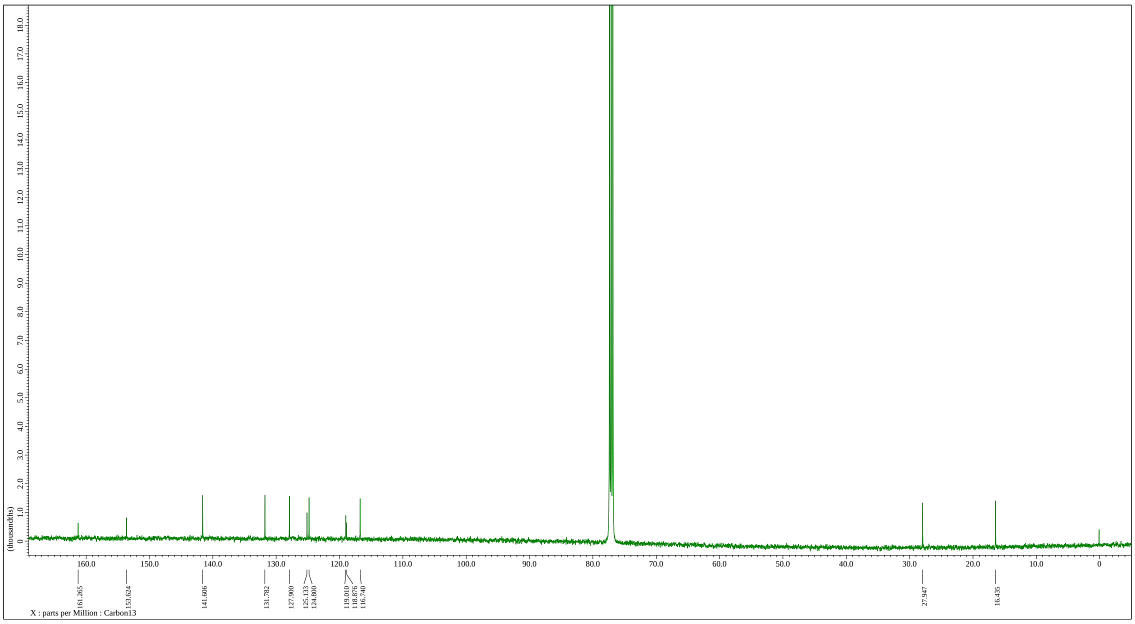

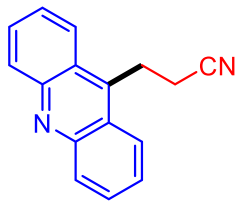

4oA

^{1}H NMR (500 MHz, $CDCl_3$)

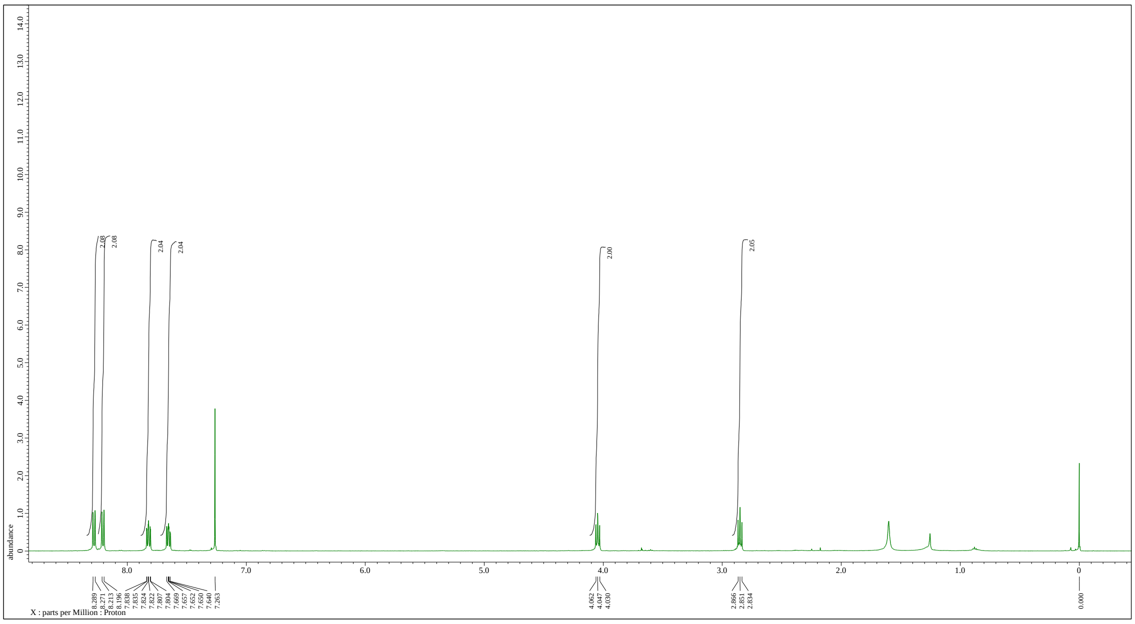

$^{13}C\{^{1}H\}$ NMR (125 MHz, $CDCl_3$)

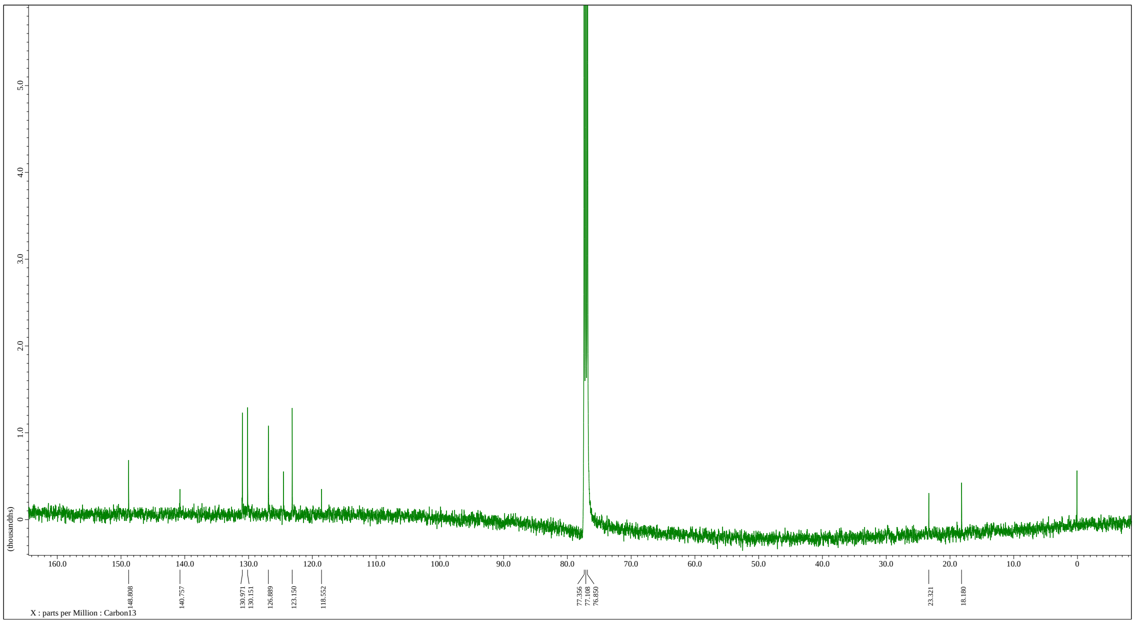

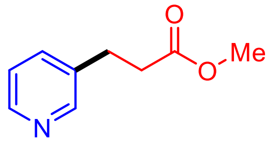

4eB

^{1}H NMR (500 MHz, $CDCl_3$)

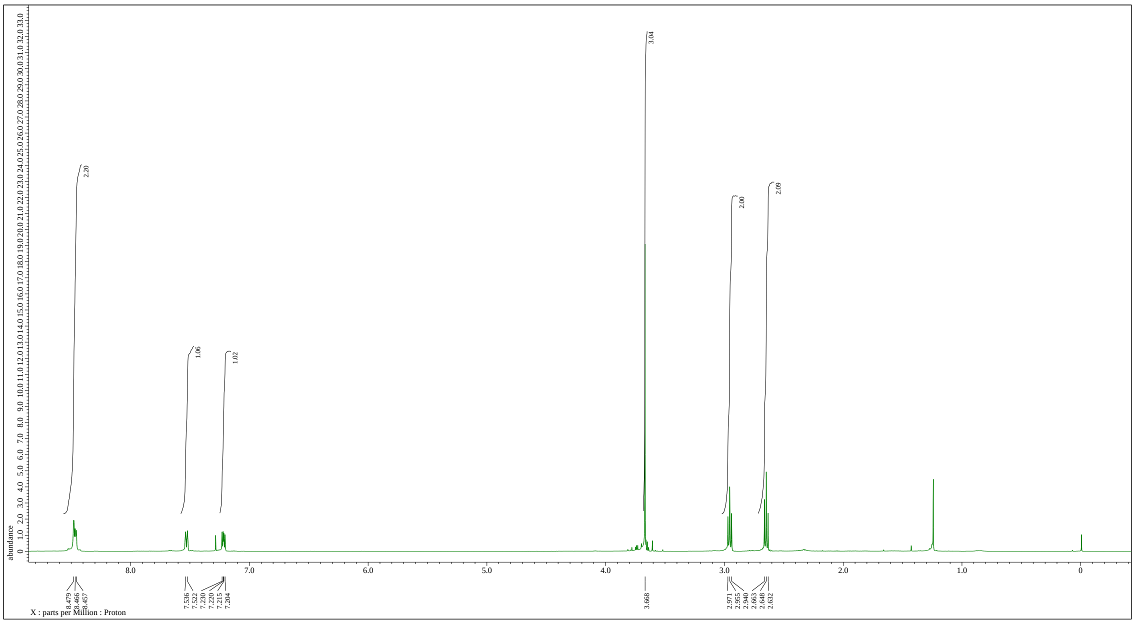

^{13}C{^{1}H} NMR (125 MHz, $CDCl_3$)

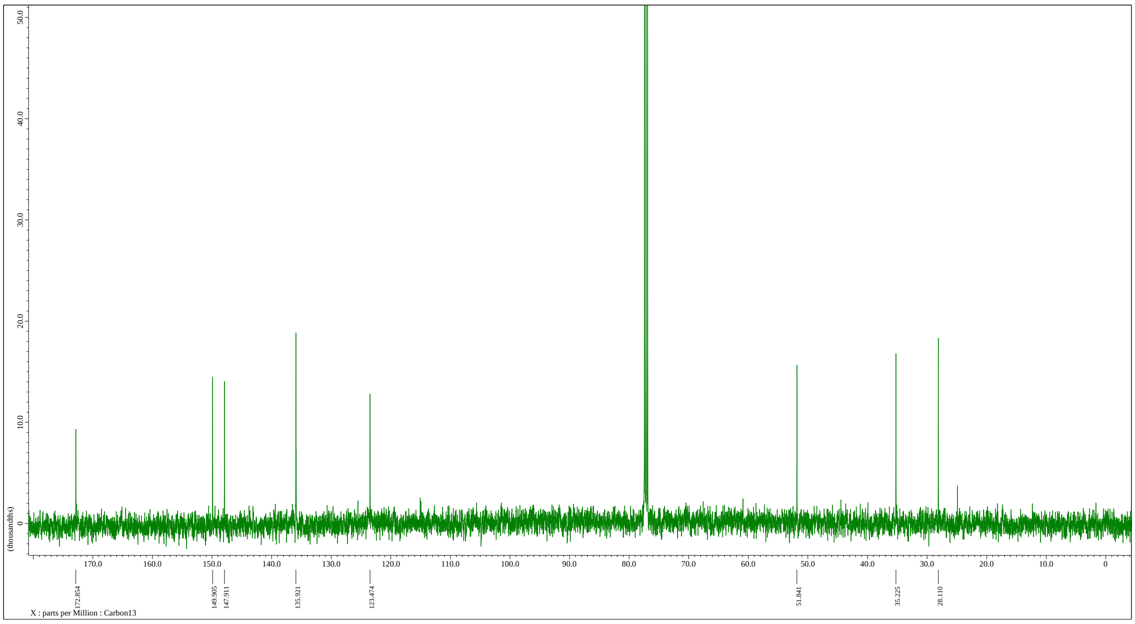

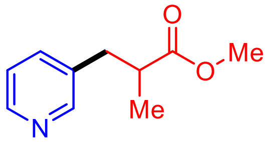

4eC

^{1}H NMR (500 MHz, $CDCl_3$)

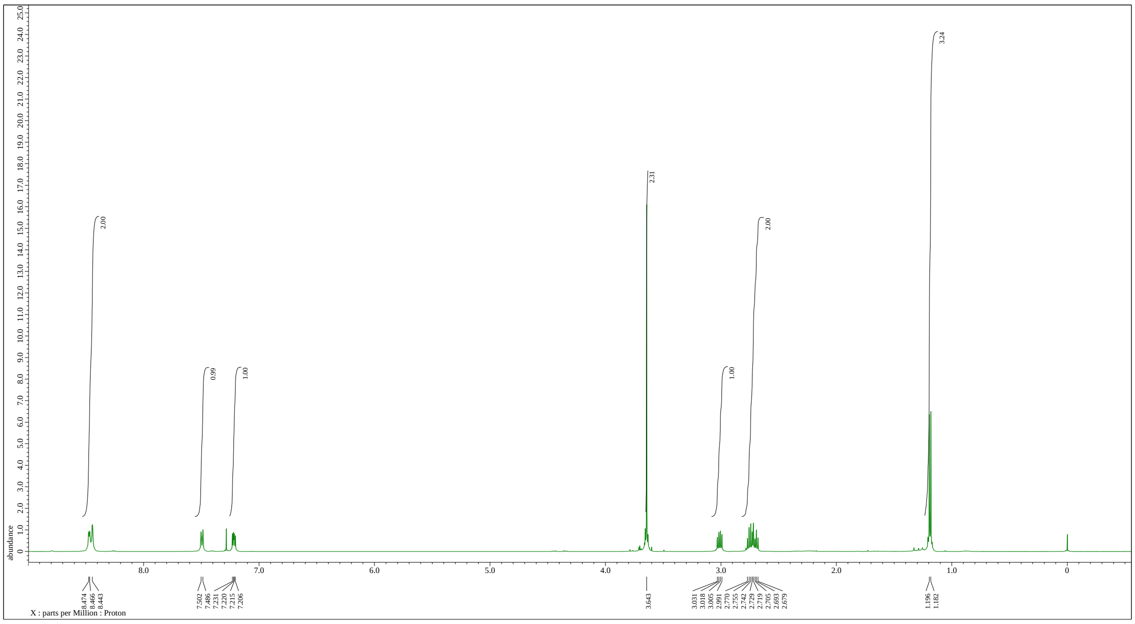

$^{13}C\{^{1}H\}$ NMR (125 MHz, $CDCl_3$)

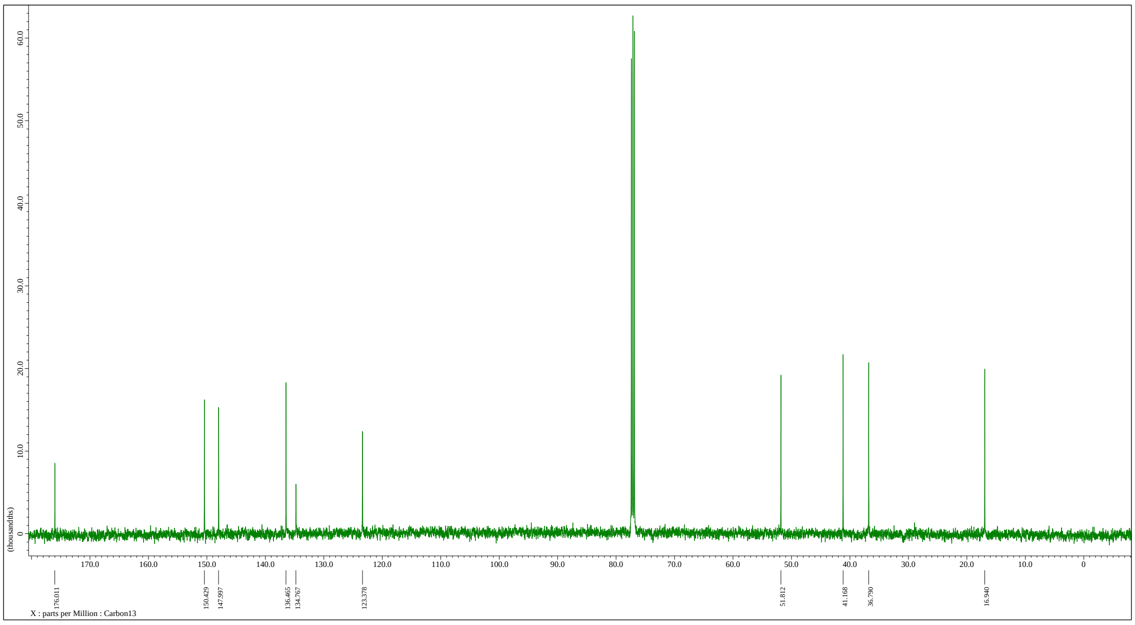

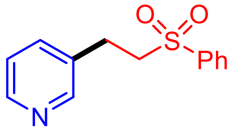

4eD

^{1}H NMR (500 MHz, $CDCl_3$)

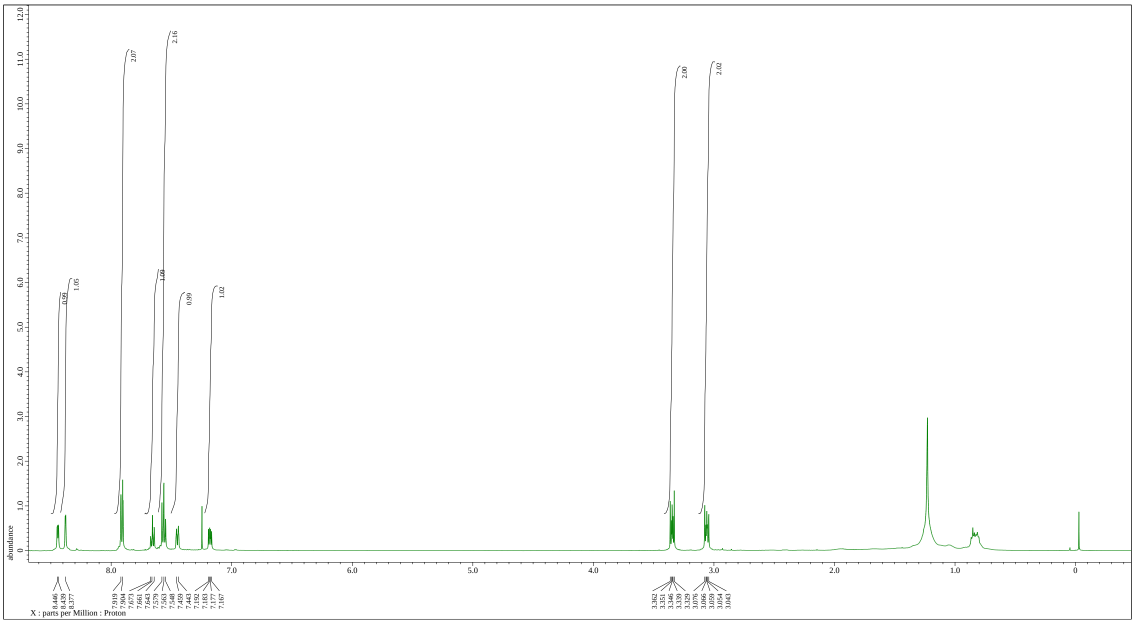

$^{13}C\{^{1}H\}$ NMR (125 MHz, $CDCl_3$)

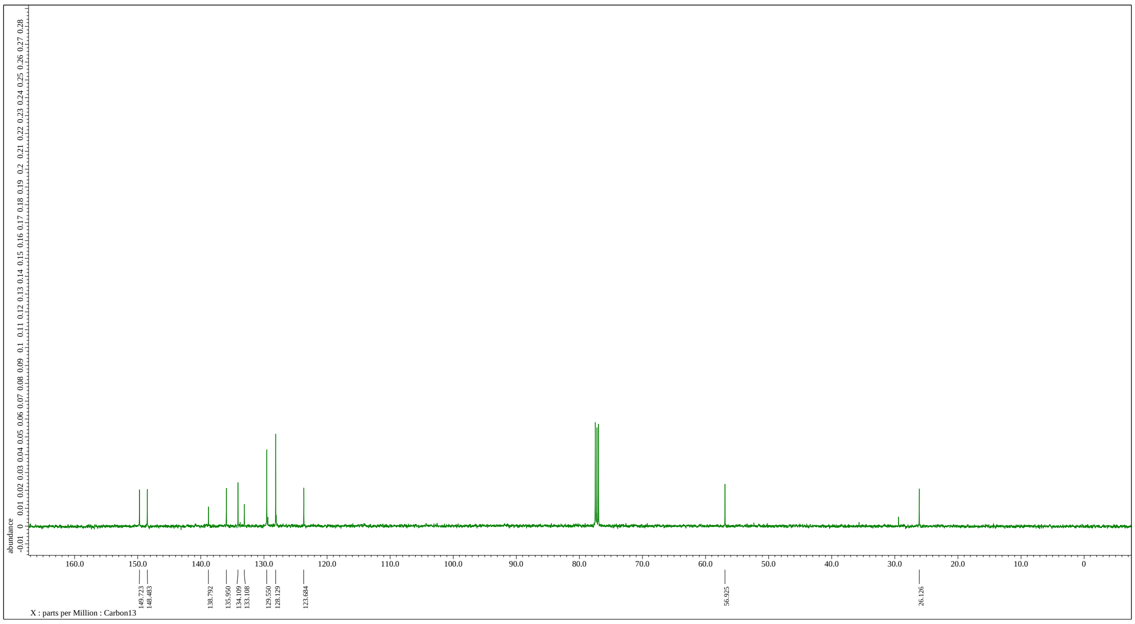

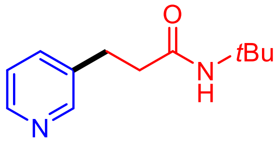

4eE

^{1}H NMR (500 MHz, $CDCl_3$)

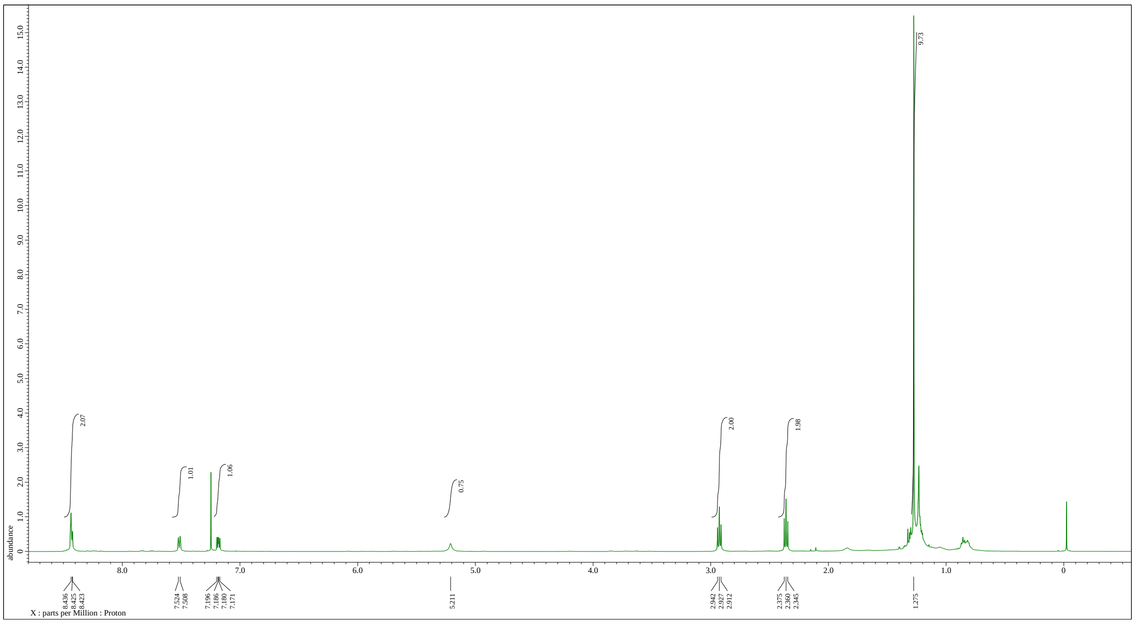

^{13}C{^{1}H} NMR (125 MHz, $CDCl_3$)

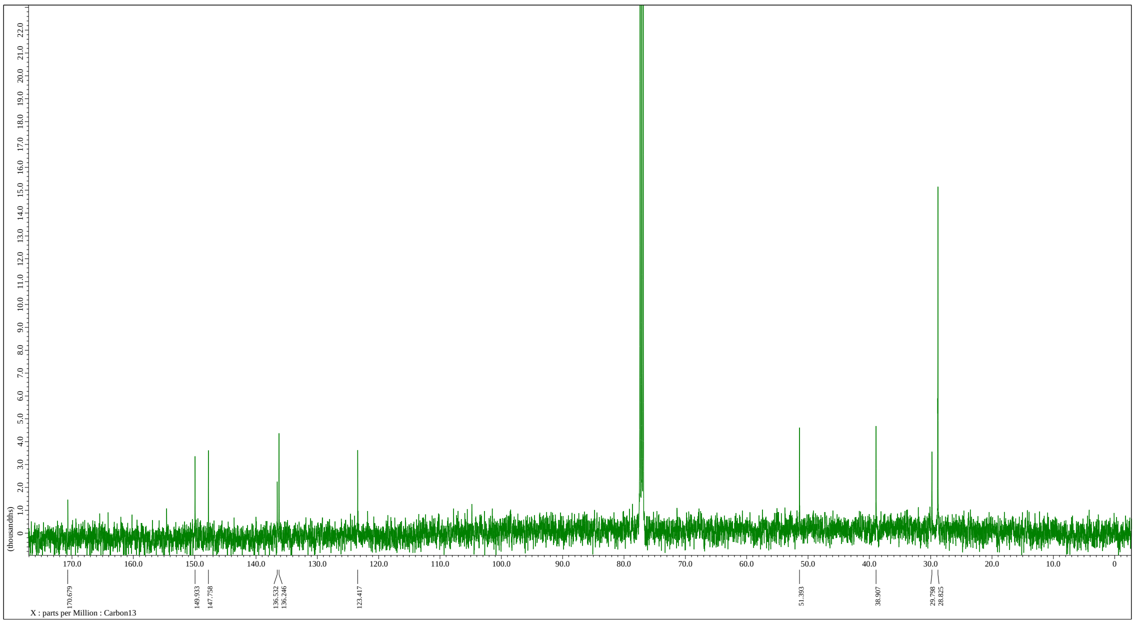

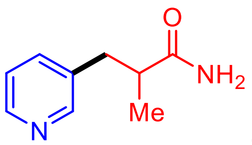

4eF

^{1}H NMR (500 MHz, $CDCl_3$)

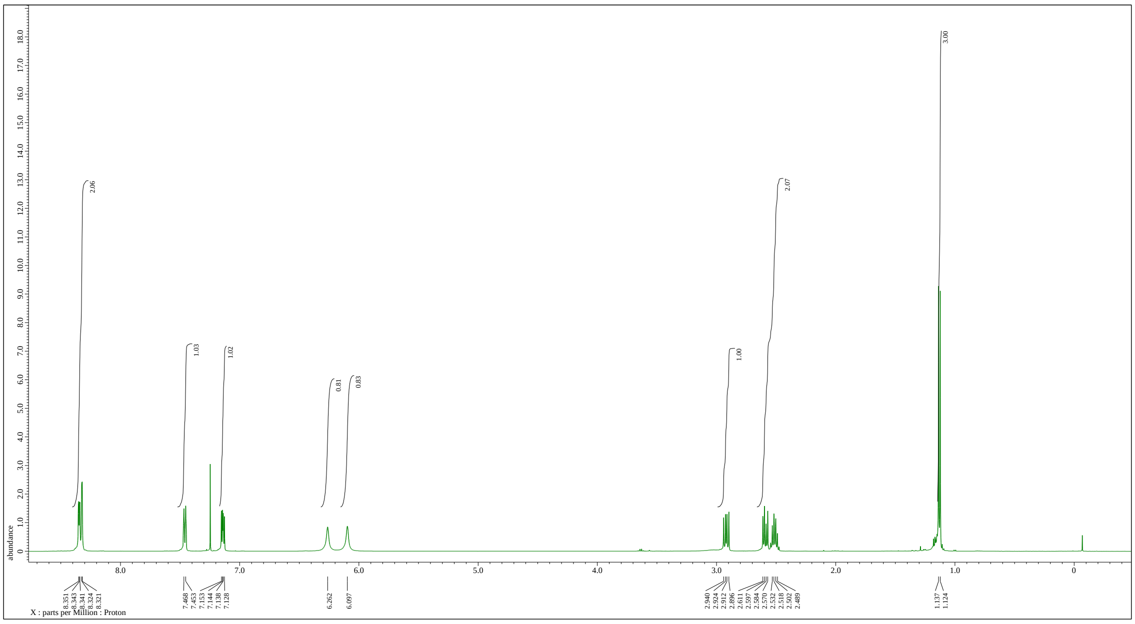

^{13}C{^{1}H} NMR (125 MHz, $CDCl_3$)

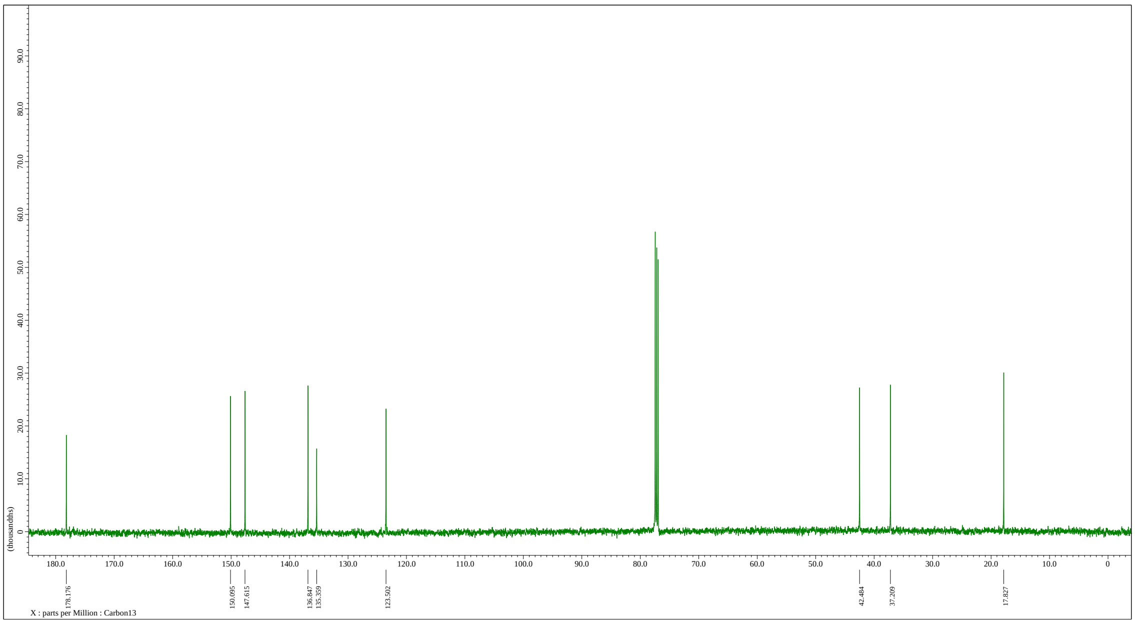

Supplement: Supplementary file 1 — Supplementary Material [file OPEN-14-e202500232-s001.pdf]
